# Lingualyzer: A computational linguistic tool for multilingual and multidimensional text analysis

**DOI:** 10.3758/s13428-023-02284-1

**Published:** 2023-11-29

**Authors:** Guido M. Linders, Max M. Louwerse

**Affiliations:** 1 Department of Cognitive Science & Artificial Intelligence, Tilburg University, Tilburg, Netherlands; 2 Department of Comparative Language Science, University of Zurich, Zurich, Switzerland

**Keywords:** Text analysis, Multilingual, Computational linguistics, Quantitative linguistics, Cross-linguistic

## Abstract

Most natural language models and tools are restricted to one language, typically English. For researchers in the behavioral sciences investigating languages other than English, and for those researchers who would like to make cross-linguistic comparisons, hardly any computational linguistic tools exist, particularly none for those researchers who lack deep computational linguistic knowledge or programming skills. Yet, for interdisciplinary researchers in a variety of fields, ranging from psycholinguistics, social psychology, cognitive psychology, education, to literary studies, there certainly is a need for such a cross-linguistic tool. In the current paper, we present Lingualyzer (https://lingualyzer.com), an easily accessible tool that analyzes text at three different text levels (sentence, paragraph, document), which includes 351 multidimensional linguistic measures that are available in 41 different languages. This paper gives an overview of Lingualyzer, categorizes its hundreds of measures, demonstrates how it distinguishes itself from other text quantification tools, explains how it can be used, and provides validations. Lingualyzer is freely accessible for scientific purposes using an intuitive and easy-to-use interface.

## Introduction

For most research in cognitive and social psychology, psycholinguistics, and cognitive science at large, text analysis has primarily focused on a very small and very specific part of human language, that of formal, written English from a WEIRD (Western, Educated, Industrialized, Rich and Democratic) population (Blasi et al., 2022; Henrich et al., 2010; Kučera & Mehl, 2022; Levisen, 2019). Most experiments conducted in these disciplines use English stimuli, and most linguistic analyses and computational linguistic tools are based on the English language. Yet, the focus on English is rather surprising. English is only one of over 7000 languages in the world, and not even the one most commonly used by native speakers (Eberhard et al., 2022). Moreover, the overwhelming focus on English might even hinder progress in these fields, due to premature generalizations across languages, based on just English-language studies (Blasi et al., 2022), and the Anglocentric bias in creating and testing hypotheses and theories (Levisen, 2019). It is therefore unlikely that all findings in the behavioral sciences can be generalized across languages (Evans & Levinson, 2009), as demonstrated in for example cross-linguistic reading experiments (Li et al., 2022; Share, 2008), cross-linguistic visual perception experiments (Lupyan et al., 2020), and analyses of backchannel behavior across languages (Maynard, 1986; Zellers, 2021). At the very least, whether findings obtained are generalizable beyond English requires an investigation to what extent, and in which ways, languages differ from one another. Because languages vary widely in their statistical regularities and cultures strongly influence the interpretation of the results of (quantitative) linguistic analyses, such analyses do not necessarily extend beyond the findings for the specific language or linguistic population under investigation (Blasi et al., 2022; Kučera & Mehl, 2022; Levisen, 2019; Louwerse, 2021).

Fortunately, there are some, albeit few, computational tools that focus on individual languages other than English. For instance, several language-specific tools have been created that can perform a large range of general natural language processing (NLP) tasks, such as word tokenization and segmentation, part-of-speech (PoS) tagging and named-entity recognition. Examples are CAMeL Tools for Arabic (Obeid et al., 2020), BNLP for Bengali (Sarker, 2021), FudanNLP for Chinese (Qiu et al., 2013), and EstNLTK for Estonian (Laur et al., 2020). However, these tools tend to be very language-specific. Extending these tools to other languages or comparing texts across different languages is difficult (Bender, 2009). The Linguistic Inquiry and Word Count (LIWC) tool, for instance, quantifies word use through a dictionary of (English) words which are grouped into primarily psychologically based dimensions (Tausczik & Pennebaker, 2010). LIWC thus heavily relies on a handcrafted dictionary that is only available in English. Attempts have been made to manually translate this dictionary into many other languages: Arabic, Brazilian Portuguese, Chinese, Dutch, French, German, Italian, Japanese, Serbian, Spanish, Romanian and Russian (see Kučera & Mehl, 2022, for an overview). However, manual translations are non-trivial and time-consuming (Boyd et al., 2022). Moreover, dictionaries in different languages vary significantly in terms of the number of words they contain (Kučera & Mehl, 2022). Perhaps more importantly, it is unclear to what extent these dictionaries are really comparable across languages. For example, a parallel corpus of TED talks was analyzed in four different languages using four different translations of the LIWC dictionary to investigate the comparability across languages (Dudău & Sava, 2021). The results varied across language pairs and even across word groups, questioning to what extent this variation can be explained by cross-linguistic differences or differences across these dictionaries.

The solution to the problem of discrepancies across languages is to not use a (top-down) dictionary approach but to rely on a (bottom-up) data-driven approach. However, data-driven tools can also be hard to extend beyond English, due to the lack of natural language training data that these tools need, and differences in annotation across languages. For example, recent neural network language models rely on very large amounts of language data. Yet data quality and quantity are both important factors in the performance of these models in different natural language processing tasks, highlighting the importance of collecting and annotating large amounts of high-quality natural language data for languages beyond English and especially for low-resource languages (Artetxe et al., 2022; Magueresse et al., 2020; Rae et al., 2021).

Fortunately, in recent years more resources for languages other than English have been made available. Most notable is the creation of the Universal Dependencies (UD) treebank collection (Nivre et al., 2020). This collection contains natural language data in many languages, annotated using a universal set of part-of-speech tags, morphological features and a universal approach to tokenization, PoS tagging, morphological feature annotation, lemmatization, dependency parsing, and named entity recognition. Several tools have been trained on the UD treebanks to automatically process and annotate new texts. Some prominent ones, covering over 60 languages, are Stanza (Qi et al., 2020) and UDPipe (Straka & Straková, 2017; Straka et al., 2016). Other resources that have been made available are the multilingual word vectors which are available in 157 languages (Grave et al., 2018), and a large-scale multilingual masked language models trained on 100 languages (Conneau et al., 2020). These resources utilize large amounts of publicly available data in many languages, such as data coming from Wikipedia and Common Crawl.

What these multilingual NLP tools lack, however, is an interface that allows users who do not have a strong background in programming and NLP to extract relevant information from (multilingual) language datasets and configure them in such a way that they can serve as measures of interest. It is exactly for that reason that quantitative text analysis tools such as LIWC (Tausczik & Pennebaker, 2010) and Coh-Metrix (Graesser et al., 2004; McNamara et al., 2014) were developed.

Quantitative text analysis converts unstructured text into quantifiable (i.e., countable or measurable) variables (Roberts, 2000), thereby leveraging the many statistical regularities that are present in human language (Gibson et al., 2019). These statistical regularities are fundamental in understanding language (Louwerse, 2011, 2018). However, these regularities are not static and differ across writers and speakers (Pennebaker & King, 1999), as well as across language registers and genres (Biber, 1988; Louwerse et al., 2004).

The quantification of language use can provide important insights in psychological processes (Linders & Louwerse, 2023), the mental state of the language user (Tausczik & Pennebaker, 2010), but in other characteristics of the language user as well, such as age and gender (Maslennikova et al., 2019; Schler et al., 2005), the idiolect and sociolect of an author (Louwerse, 2004), the native language of a writer (Malmasi et al., 2017), and even demographic information (Alvero et al., 2021). What’s more, the regularities in language are different enough between different language users such that it is possible to identify the author of a piece of text (Juola, 2008; Türkoğlu et al., 2007). Quantitative text analysis is also used for stimulus creation (Cruz Neri & Retelsdorf, 2022a), validation (Trevisan & García, 2019) and analysis (Dodell-Feder et al., 2011). Finally, the resulting quantification is used in computational and statistical models to infer and understand latent properties of texts, such as the truth value of political statements (Mihalcea & Strapparava, 2009; Rashkin et al., 2017), whether social media texts contains humor or irony (Barbieri & Saggion, 2014; Reyes et al., 2012) or hate speech (Fortuna & Nunes, 2018), and the readability of a text (McNamara et al., 2012). In sum, there is a variety of research purposes for which quantitative text analysis tools are desirable.

To support multilingual and multidimensional text analysis, we created the computational linguistic tool Lingualyzer (https://lingualyzer.com). Specifically, we had four goals in mind. First, the tool had to support languages beyond English and beyond the Indo-European language family (as many languages as were feasible), and allow for comparable output in all languages. Second, the tool had to be accessible for researchers that do not necessarily have knowledge of NLP or programming. Concretely, this meant providing users with an interface where they can enter unstructured and unprocessed text and with a few clicks obtain the values for a large number of different measures at different text levels (i.e., sentence, paragraph, and document) and linguistic dimensions (e.g., lexical, syntactic, and semantic dimensions). Third, we strived to include a large and varied set of reliable linguistic dimensions and linguistic measures to maximize the value of the tool for different purposes (e.g., cross-linguistic comparisons, text characterization, stimulus validation). Fourth, the linguistic features included in the tool needed to be motivated theoretically. Finally, to make the tool readily available, we aimed for a web interface, freely accessible for scientific purposes.

The current paper is structured into three parts. First, we provide an overview of the existing tools in the literature to position Lingualyzer. Next, we present an overview of Lingualyzer and the linguistic measures and dimensions it covers. Finally, we provide an evaluation of Lingualyzer in terms of instrument reliability and instrument validity.

### Tools for a quantitative text analysis

What text analysis tools are already available for researchers working in the behavioral sciences? It is difficult to provide an exhaustive overview of existing tools, given the variety in measures and the focus of the available tools, the variety of single languages they cover, and the variety of publications in journals from different disciplines and in different languages (other than English). Without aiming for an exhaustive overview, but rather to get a general idea of the variety of the available quantitative text analysis tools, we provide an overview whereby we restricted ourselves to only include those tools (1) that had a clear focus on quantitative text analysis, (2) that covered the whole processing pipeline from processing the raw, unstructured text to the quantitative analysis, (3) with an interface accessible to the user, rather than the developer of the tools, (4) that were not derivatives or subsets of other tools, for example tools that were translated into other languages (e.g., Scarton & Aluísio, 2010; Van Wissen & Boot, 2017), and (5) that had more than three measures, to exclude tools that focus on a single natural language processing or text classification task (e.g., Thelwall et al., 2010).

An overview of quantitative text analysis tools is given in Table 1. The foci of these tools vary. Some focus on text characterization to measure the variation in language use with respect to different populations (Brunato et al., 2020; Francis & Pennebaker, 1992; McTavish & Pirro, 1990), language registers (Biber, 1988, as reimplemented in Nini, 2019) or reflecting aspects of cognition (Tuckute et al., 2022). Others focus on specific text characteristics, such as sentiment and verbal tone (Crossley et al., 2017; North et al., 1972). Yet, other tools focus on text complexity in terms of readability (Bengoetxea & Gonzales-Dios, 2021; Dascalu et al., 2013), text cohesion (Crossley et al., 2016; Dascalu et al., 2013; Graesser et al., 2004) or syntactic complexity (Kyle, 2016; Lu, 2010). It is, however, important to note that tools can be used for multiple purposes. For example, T-Scan (Pander Maat et al., 2014) and LATIC (Cruz Neri et al., 2022b) have been designed to quantify both text characteristics and complexity, while Coh-Metrix, for example has been used to characterize variation in language registers (Louwerse et al., 2004) and authorship attribution (McCarthy et al., 2006).

**Table 1:** Overview of existing quantitative text analysis tools

Name	Focus	Approach	No. measures included	Languages and register focus	Locally running or online available	Conditions on accessibility	Earliest reference	Reference of most recent version
Diction	Verbal tone	Dictionary-based	5	English	Local	Everyone	North et al. (1972)	Hart (2017)
Biber Tagger	Register variation	Dictionary-based	67	English	Local	Experts	Biber (1988)	Nini (2019)
DIMAP-MCCA	Linguistic profiling	Dictionary-based	± 150	English	Local	Everyone	McTavish & Pirro (1990)	
LIWC	Linguistic profiling	Dictionary-based	± 90	English	Local	Everyone	Francis & Pennebaker (1992)	Boyd et al. (2022)
Coh-Metrix	Text cohesion	Data-driven	108–200	Written English	Local	Everyone	Graesser et al. (2004)	McNamara et al. (2014)
L2SCA	Syntactic complexity	Data-driven	14	Written English as a second language	Both	Experts	Lu (2010)	
ReaderBench	Text cohesion	Data-driven	± 330	Dutch, English, French, German, Italian, Romanian, Russian and Spanish	Both	Everyone	Dascalu et al. (2013)	Gutu-Robu et al. (2018)
T-Scan	Language variation and text complexity	Hybrid	472	Dutch	Both	Everyone	Pander Maat et al. (2014)	
TAACO	Text cohesion	Data-driven	150–194	Written English	Local	Experts	Crossley et al. (2016)	Crossley et al., (2019)
TAASSC	Syntactic complexity	Data-driven	372	Written English as a second language	Local	Experts	Kyle (2016)	
SEANCE	Sentiment	Hybrid	270	English	Local	Experts	Crossley et al. (2017)	
Profiling-UD	Linguistic profiling	Data-driven	87–130	59 languages	Online	Everyone	Brunato et al. (2020)	
MultiAzterTest	Readability	Data-driven	125–163^1^	Written Basque, English and Spanish	Online	Everyone	Bengoetxea & Gonzales-Dios (2021)	
LATIC	Text characteristics and readability	Data-driven	43	English, French, German, and Spanish	Local	Everyone	Cruz Neri et al. (2022b)	
SentSpace	Cognitive processes	Hybrid	± 30	English sentences	Both	Everyone	Tuckute et al. (2022)	

Ranges and approximations of measures due to discrepancies between what is mentioned in the literature and what is available online.

^1^The number of measures differs in each language with 125 available measures in Basque, 141 in Spanish, and 163 in English.

Table 1 marks whether the approach of the given tools is primarily dictionary-based or data-driven. Dictionary-based tools are those that include a dictionary or database to categorize words and consequently categorize a text. Data-driven methods instead rely on patterns in the text and quantify those using computational linguistic and statistical models. Hybrid approaches use a mixture of both dictionary-based and data-driven approaches. In general, more recently developed tools tend to be more data-driven or hybrid, whereas the inception of older tools tend to be more dictionary-based. While it is difficult to create a dictionary-based tool in multiple languages because individual dictionaries or databases need to be constructed for each language, it is at the same time difficult to create data-driven tools in multiple languages because it would require natural language data that is annotated in a unified manner across the different languages.

The number of measures in Table 1 indicate the different number of quantifiable values or linguistic variables that the tool measures. These vary widely between different tools, ranging from five (Diction) to approximately 472 (T-Scan). Most tools contain between 50 and 200 measures. A comparison of absolute numbers between tools is not very meaningful, because the measures widely differ in complexity, ranging from simple word or word group counts to average semantic similarity scores of adjacent paragraphs.

Table 1 furthermore marks the languages supported by the original version of each tool. Note that this does not include any separate translations of the original tools or derivative tools that use part of the measures from the original tool. Most tools only support English, and some are even more specific, focusing only on written English (Crossley et al., 2016) or even written English as a second language (Kyle, 2016; Lu, 2010). There are, however, some tools that cover more than just the English language (Bengoetxea & Gonzales-Dios, 2021; Brunato et al., 2020; Cruz Neri et al., 2022b; Dascalu et al., 2013). These tools may support one other language, but with the exception of Profiling-UD (Brunato et al., 2020), the languages beyond English are only supported by a subset of the measures. Moreover, due to differences in annotation algorithms and tagsets being used for the different languages, it is virtually impossible to compare the output of the overlapping measures across the languages.

The tools in Table 1 are ordered by the year the first version was released. Many tools have seen improvements over time, and as such, we have also added the most recent reference that provides information on the most recent changes or additions.

## Lingualyzer

Lingualyzer is a multilingual and multidimensional text analysis tool available for scientific purposes, benefiting behavioral science researchers who may not have a strong NLP programming background or otherwise would like to use an easily accessible tool. Lingualyzer computes a large number of linguistic measures across different dimensions and levels of analysis. This section explains Lingualyzer, starting with an overview of the languages for which it is available. We then outline how Lingualyzer processes texts and give an overview of the different dimensions that Lingualyzer captures. We end this section with an explanation of how Lingualyzer can be used.

### Languages

Table 2 summarizes all 41 languages and their respective ten language families for which Lingualyzer is currently available. Within the Indo-European family alone, the tool covers seven different branches. One of the core principles of Lingualyzer is a uniform treatment of text regardless of its language. This means that all measures are available in all languages. Consequently, all values are calculated using exactly the same computations, and annotations are performed based on the same strategies and schemes, which is not the case for most other multilingual quantitative text analysis tools, such as ReaderBench (Dascalu et al., 2013), MultiAzterTest (Bengoetxea & Gonzales-Dios, 2021) and LATIC (Cruz Neri et al., 2022b). This means that the output of all the measures are comparable across languages.

**Table 2:** Overview of the 41 languages that Lingualyzer supports

Table 2a		Table 2b	
Language	Family	Family	Language
Afrikaans	Indo-European (Germanic)	Afro-Asiatic	Arabic
Arabic	Afro-Asiatic	Afro-Asiatic	Hebrew
Catalan	Indo-European (Romance)	Austro-Asiatic	Vietnamese
Chinese	Sino-Tibetan	Austronesian	Indonesian
Croatian	Indo-European (Slavic)	Dravidian	Telugu
Czech	Indo-European (Slavic)	Indo-European (Baltic)	Latvian
Danish	Indo-European (Germanic)	Indo-European (Baltic)	Lithuanian
Dutch	Indo-European (Germanic)	Indo-European (Celtic)	Welsh
English	Indo-European (Germanic)	Indo-European (Germanic)	Afrikaans
Estonian	Uralic	Indo-European (Germanic)	Danish
Finnish	Uralic	Indo-European (Germanic)	Dutch
French	Indo-European (Romance)	Indo-European (Germanic)	English
German	Indo-European (Germanic)	Indo-European (Germanic)	German
Greek	Indo-European (Greek)	Indo-European (Germanic)	Icelandic
Hebrew	Afro-Asiatic	Indo-European (Germanic)	Norwegian
Hindi	Indo-European (Indo-Iranian)	Indo-European (Germanic)	Swedish
Hungarian	Uralic	Indo-European (Greek)	Greek
Icelandic	Indo-European (Germanic)	Indo-European (Indo-Iranian)	Hindi
Indonesian	Austronesian	Indo-European (Indo-Iranian)	Persian
Italian	Indo-European (Romance)	Indo-European (Indo-Iranian)	Urdu
Japanese	Japonic	Indo-European (Romance)	Catalan
Korean	Koreanic	Indo-European (Romance)	French
Latvian	Indo-European (Baltic)	Indo-European (Romance)	Italian
Lithuanian	Indo-European (Baltic)	Indo-European (Romance)	Portuguese
Norwegian	Indo-European (Germanic)	Indo-European (Romance)	Romanian
Persian	Indo-European (Indo-Iranian)	Indo-European (Romance)	Spanish
Polish	Indo-European (Slavic)	Indo-European (Slavic)	Croatian
Portuguese	Indo-European (Romance)	Indo-European (Slavic)	Czech
Romanian	Indo-European (Romance)	Indo-European (Slavic)	Polish
Russian	Indo-European (Slavic)	Indo-European (Slavic)	Russian
Serbian (Latin)	Indo-European (Slavic)	Indo-European (Slavic)	Serbian (Latin)
Slovak	Indo-European (Slavic)	Indo-European (Slavic)	Slovak
Slovenian	Indo-European (Slavic)	Indo-European (Slavic)	Slovenian
Spanish	Indo-European (Romance)	Indo-European (Slavic)	Ukrainian
Swedish	Indo-European (Germanic)	Japonic	Japanese
Telugu	Dravidian	Koreanic	Korean
Turkish	Turkic	Sino-Tibetan	Chinese
Ukrainian	Indo-European (Slavic)	Turkic	Turkish
Urdu	Indo-European (Indo-Iranian)	Uralic	Estonian
Welsh	Indo-European (Celtic)	Uralic	Finnish
Vietnamese	Austro-Asiatic	Uralic	Hungarian

Overview of the 41 languages included in Lingualyzer ordered by language (Table 2a) and by language family (Table 2b).

### Natural language processing resources

For a multilingual text analysis tool that can be used for cross-linguistic analyses, consistency across languages is important. Text processing therefore needs to be unified across languages, using an NLP pipeline that covers a diversity of languages. Fortunately, two such NLP pipelines that facilitate this process already exist: Stanza (Qi et al., 2020) and UDPipe (Straka & Straková, 2017). Both Stanza and UDPipe are open-source toolkits available in Python that can perform many different natural language processing tasks on raw texts, including word tokenization, lemmatization, PoS tagging, named entity recognition, and dependency parsing and were developed with the goal of creating a language-agnostic tool that is available in as many language as possible (currently over 60). The models of both toolkits are trained on the Universal Dependencies (UD) treebanks (Nivre et al., 2020) and come with a framework, such that with relative ease, new models can be trained in new languages.

Despite their similarities, there are differences between the two frameworks that makes Stanza preferable. Most importantly, Stanza makes use of deep neural network models to reach a state-of-the-art performance on the core natural language processing tasks, whereas UDPipe makes use of both deep neural networks and machine learning methods, reaching a very similar, but generally slightly lower performance compared with Stanza on all NLP tasks (cf. Qi et al., 2020). Moreover, because UDPipe is slightly older, its models were trained on an older version of the UD treebanks.

The UD framework provides a universal approach to annotating texts on different morphosyntactic levels. First, word boundaries are determined. Words are then annotated with a lemma, a bare form of the word with all morphology removed, and with a syntactic label. The UD framework makes use of a universal set of 17 different PoS tags, which can be summarized into open class tags (i.e., adjectives, adverbs, interjections, nouns, proper nouns and verbs), closed class tags (i.e., adpositions, auxiliaries, coordinating and subordinating conjunctions, determiners, numerals, particles, and pronouns) and a group of other tags, including punctuation markers, symbols and a rest category. Finally, words are annotated with one or multiple morphosyntactic properties. These are labels that indicate different lexical and grammatical features that are overtly marked on the words. The UD framework supports 24 different classes which are further subdivided into individual features. Features are annotated using a presence value, such that a word either does contain a certain feature or does not. Because not all features are present or annotated in every language, we have made a selection of the most universal features and included those in Lingualyzer. These include personal, demonstrative, and interrogative pronouns, singular and plural words, definite and indefinite words, finite verbs, infinitives and verbal adjectives, present and past tense markers, and passive voice markers.

Importantly, the UD framework is word- or token-based, with all lemmas, part-of-speech tags, morphosyntactic properties annotated on a word level. Consequently, Stanza is also word-based, and in turn Lingualyzer quantifies most units on a word or token level. In most languages words and tokens are identical and can be identified by whitespaces around the word. There are, however, two exceptions. First, there are languages such as Chinese, Japanese, and Vietnamese, that do not use whitespaces to mark word boundaries.^1^ In these cases, Stanza uses a word segmentation algorithm to decide the word boundaries. Words and tokens are therefore still identical, but they cannot be directly observed from the text through whitespace boundaries. Word segmentation is also used in other languages, albeit on a much smaller scale. For example in English, possessive markers (marked by *’s*) are seen as separate word tokens by Stanza and some compound words, such as *government-friendly* are split into two separate word tokens. Second, some languages such as French, Italian, and Spanish, use contractions or other mechanisms to represent multiple words into a single word that is bounded by whitespaces. In such languages, Stanza requires by default a multiword expression token identifier (Qi et al., 2020). In such cases, words and tokens will differ from each other, since each multiword comprises multiple tokens. Each token, instead of each word, in those cases will be annotated with a lemma, a PoS tag and morphosyntactic features. Note that this differs from the segmentation of for example compound words in English, where each token in the compound is seen as a separate word, and hence where no distinction between words and tokens is made.

Even though Stanza is a useful tool on its own, it is not directly accessible for behavioral science researchers with no or limited background in NLP or programming. Moreover, Stanza does not provide insights into different linguistic dimensions. Lingualyzer is therefore not a copy of Stanza but computes a large set of measures *based on* the Stanza tool in order to quantify text (sentence, paragraph, document) on a wide variety of linguistic dimensions. In other words, Stanza provides the linguistic annotations, such as tokenization and PoS tagging, that Lingualyzer then uses to compute different measures that give insights in different linguistic dimensions.

We enhanced the processed text from Stanza with two other language-specific sources to maximize the dimensions of text analysis: word vectors and a database of standardized word frequencies. Word vectors contain valuable information on the distributional properties of words. We used the 300 dimensional fastText word vectors, which were created from Wikipedia and Common Crawl data (Grave et al., 2018). These word vectors are trained on character *n*-grams and are therefore also able to generate an approximate vector representation for words for which no word vector is already stored. However, storing the trained word vector model in memory for each language is not feasible because of the large size of the models and the many languages in Lingualyzer. We therefore opted for storing all word vectors for complete words in a database and decided not to approximate vectors for words that are not in the database.

Standardized word frequencies provide valuable information on the general use of words beyond the text under investigation. We used the frequency lists from WorldLex for each available language (Gimenes & New, 2016). These frequency lists are based on three different large language sources: news articles, blogs, and Twitter data. Because the Twitter data were not available for all languages, we decided not to use it to maintain consistency across languages. For the remaining sources, we had absolute and normalized frequencies and contextual diversity measures to our availability. We, however, only used the normalized values, which represent the frequency per million words and the percentage of documents a word occurred in, respectively. The frequency lists for each source contained a minimum of 1.8 million words and 41,000 documents. We removed all words in the list that did not reach a threshold frequency of once per million words to mitigate any effects the size of the frequency lists, and to subsequently keep the quantification steps across language entirely similar and unbiased. Hence, the advantage of having a standardized list with a frequency threshold is that the lists, and in turn the output of the measures using these lists, are comparable across languages.

### Levels of analysis

A text can be described as a complex collection of smaller linguistic segments, from morphological units, to words, sentences, and paragraphs, to entire documents. We implemented three different levels of analysis on which a text can be analyzed: the sentence level, the paragraph level, and the document level. Because sentence boundaries are denoted differently across languages, Stanza is used here for their identification. Paragraphs on the other hand are identified through a double newline separator in the document.

Importantly, all measures can be computed on each of those three levels, thus making no distinction in how the value for each measure is calculated based on these levels. However, since a value is calculated for each paragraph or sentence, returning each value individually is not feasible, nor desirable, since the number of values returned to the user would then be dependent on the text size and would not be uniform across analyses. For this reason, Lingualyzer summarizes the values for the paragraph and sentence levels using different statistics. More specifically, the values for the paragraph and sentence levels are summarized into average values over the different paragraphs or sentences, the standard deviation from this average, and the largest (maximum) and smallest value (minimum) across paragraphs or sentences.

### Overview of Lingualyzer measures

**Fig. 1:**
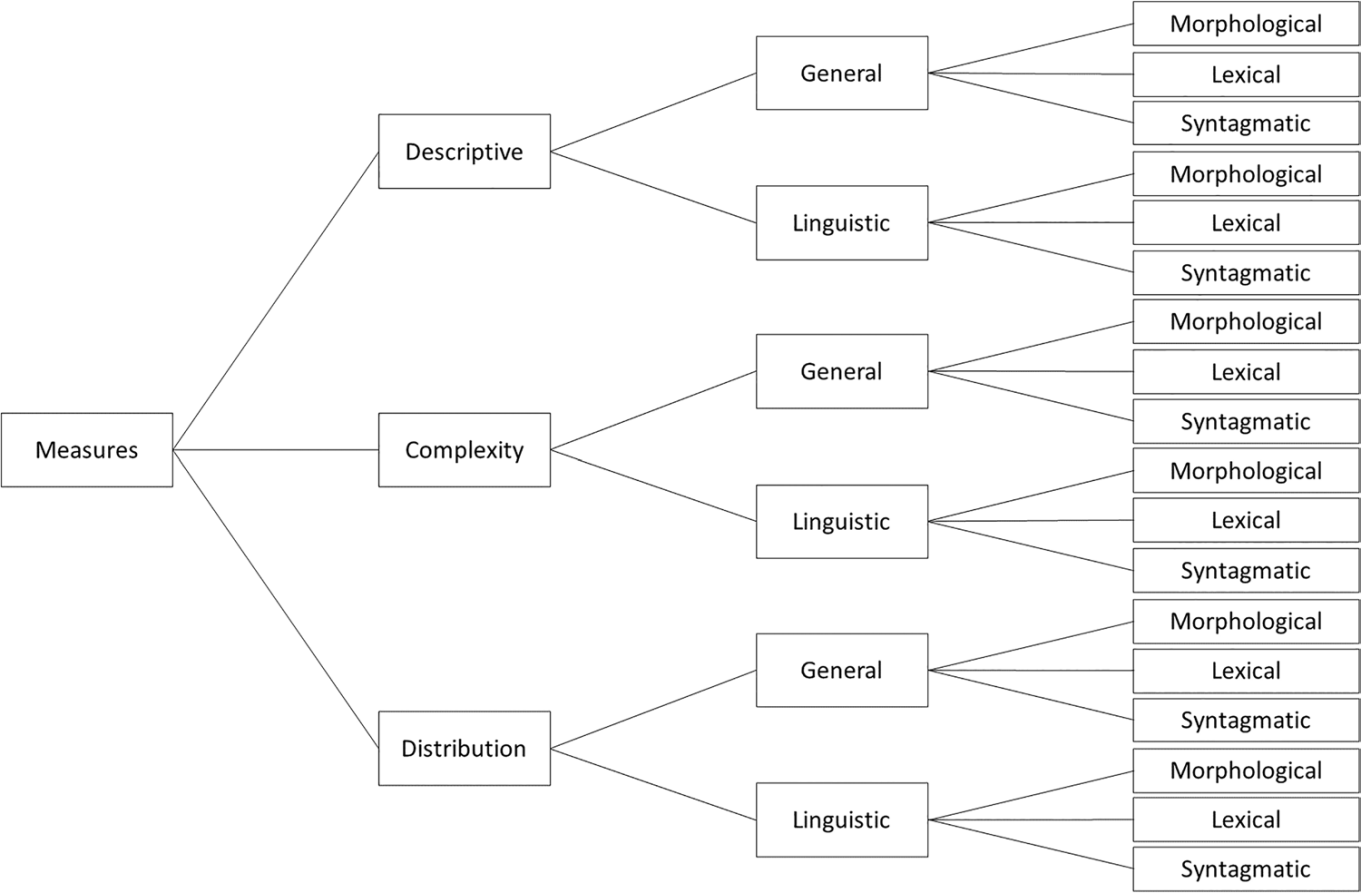
Categorization of measures

Due to the large number of measures, it is impossible within the scope of this paper to describe each Lingualyzer measure individually. Instead, below we categorize the 351 measures (Fig. 1) and provide a description of the individual measures in the instructions in the online interface. A categorization of measures needs to be independent of the text segment (i.e., sentence, paragraph or document) being analyzed. Furthermore, a categorization based on just the calculation method that is used to determine value of the measure (e.g., count or ratio) does not suffice, because in many cases the same calculation method can be applied to many different aspects of a text, resulting in categories that are not necessarily very meaningful.

Based on the type of information captured, we distinguish three categories of measures: (1) descriptive measures, (2) complexity measures, and (3) distribution measures. Descriptive measures describe the surface level or directly observable patterns in a text segment. Complexity measures, on the other hand, target the variability or internal complexity of a text segment. These measures can also describe the relationship between different descriptive measures within a text segment. Distribution measures capture the temporal aspects of a text segment. At a non-linguistic level, these measures describe the temporal distribution of an aspect, while at the linguistic level, these measures describe the distributional relationships between different text segments.

The descriptive, complexity, and distributional measures can be subdivided into whether or not they are language-specific. If the measure is not dependent on language-specific annotations such as the PoS tag, lemma, morphological features, or frequency and word vector databases, it is considered to be *general*, otherwise the measure is labeled *linguistic*.

The resulting six (descriptive, complexity and distribution measures × general and linguistic measures) can be further subdivided into different text units quantified by the measures. We have defined measures quantifying (1) morphological, (2) lexical, and (3) syntagmatic units. Morphological measures capture patterns within the boundaries of individual words, such as morphemes or characters. Lexical measures quantify the individual words themselves. Finally, syntagmatic measures capture patterns in groups of words that share a (morpho)syntactic or semantic feature, such as a PoS tag or plural words. Note that this categorization is independent from the text level (i.e., sentence, paragraph, document) being investigated, as all morphological, lexical, and syntagmatic measures can be computed on each of these three text levels.

Altogether the Lingualyzer taxonomy encompasses (3 × 2 × 3 = ) 18 categories. The taxonomy is illustrated in Fig. 1, with the categories being discussed in more detail next.

#### Measures by information type and language-specificity

##### General descriptive measures

General descriptive measures describe the composition or the surface level characterization of a text for which no linguistic knowledge is required. They may serve as proxies for other measures, but deviate from them in that they are directly observable in a text and language independent.^2^ Examples of general descriptive measures are the letter and word count, measuring the total number of letters and words in a text segment respectively and the hapax legomena incidence, which counts the number of words occurring only once in a text per 1000 words.

##### Linguistic descriptive measures

Linguistic descriptive measures describe the surface level observable linguistic patterns. Linguistic descriptive measures are not necessarily generalizable, and hence language dependent in the sense that they require language-specific algorithms or resources to extract the required information. Examples of linguistic descriptive measures are the counts of individual part-of-speech tags (e.g., nouns, verbs, adverbs) in a text segment, or morphosyntactic features, such as the number of definite words and the number of passive voice markers in a text segment.

##### General complexity measures

General complexity measures compute the level of variability or internal complexity of a variable in terms of cognitive or computational resources, independent of a language. While general descriptive measures only describe the surface level, complexity measures look at aspects beyond, targeting latent variables of a text. Examples of general complexity measures are for instance the type-token ratio, i.e., the number of distinct words in the text compared to the total number of words, sentence length, i.e., the average number of words in a sentence, and word entropy, i.e., the average information content of the word types.

##### Linguistic complexity measures

Linguistic complexity measures compute the variability or complexity of variables in terms of linguistic variation and structure. Differing from the general complexity measures, these measures target language-specific or linguistic aspects, thus describing the variability between different linguistic markers or the complexity of the linguistic structure. Different than the linguistic descriptive measures, linguistic complexity measures describe the latent or internal structure of linguistic aspects, rather than surface level or directly observable linguistic aspects. Note that because these aspects are language-specific, there might be substantial variation in the internal linguistic structures of different linguistic variables across languages. Most notably, some linguistic aspects can be completely unmarked in a language, such that no linguistic structure is present at all. For example, definiteness is not marked in Chinese and Russian. Examples of measures in this category are the first-to-third pronoun ratio and the definite-indefinite word ratio.

##### General distribution measures

General distribution measures describe the temporal patterns in the surface level aspects of a text segment. These measures differ from the general descriptive measures because they investigate specifically *where* a surface level aspect of a text occurs, rather than describing a descriptive property of that aspect. General distribution measures furthermore differ from the general complexity measures because they investigate the surface level aspects of a text, rather than the latent aspects or internal complexity. Note that these measures also describe the surface level temporal patterns of different linguistic markers. Even though linguistic or language-specific information is needed to determine where those markers are, no linguistic information is needed for describing their temporal patterns. Hence, we have classified these as general distribution measures. Examples of such measures are the first-person pronoun burstiness, measuring the interval distribution of first-person pronouns, and the average position of future tense markers in a text segment.

##### Linguistic distribution measures

Linguistic distribution measures describe the temporal relationships between different text segments, thereby extending beyond the analysis of an individual text segment. What sets these measures apart from the general distribution measures is that they do not describe the temporal patterns within a text segment, but between different text segments, thereby describing the distributional relationships between different text segments. Whereas linguistic complexity measures describe the linguistic structure within a text segment, the linguistic distribution measures describe the similarities and differences in linguistic structure between text segments. We can compare consecutive paragraphs (paragraph-paragraph) or sentences (sentence-sentence) and calculate the values of the linguistic distribution measures for each comparison. Moreover, because we treat each text segment similarly, regardless of whether it is a document, paragraph, or sentence, we can calculate the linguistic distribution measures between text segments of different levels. Hence, we can compare each sentence to the rest of the document (sentence-document), or to the rest of the paragraph (sentence-paragraph) it occurs in, as well as compare each paragraph to the rest of the document (paragraph-document). Examples of such measures are the average word vector cosine similarity that measures the semantic similarity between two text segments. Another example is the lemma overlap between two text segments, which measures the proportion of lemmas in the smaller text segment that also occurs in the larger segment.

#### Measures by linguistic unit

For all six categories (i.e., descriptive, complexity and distribution, each subdivided into general and linguistic) we identify three additional categories of measures. These measures target different units in the text, namely within the word boundaries (morphological), at word level (lexical) and within a group of words that share a syntactic or semantic characteristic (syntagmatic).

##### Morphological measures

Morphological measures target information quantified in the word form, thus describing patterns that occur typically within the boundaries of individual words. One example is letter entropy, which measures the average information content of letters. Another example is the Levenshtein distance, which measures the distance between two text segments in terms of how many letter substitutions, additions and deletions are minimally needed to transform one text segment to the other.

##### Lexical measures

Lexical measures target information quantified at the level of individual word tokens, describing the composition, complexity or distribution of words, where individual words are the quantified units. These measures specifically target properties that are unique to a word, and thus do not target syntactic or semantic properties. Examples are the word count, hapax legomena count (number of words occurring only once in the text segment), type-token ratio, unknown word count (words not occurring in the standardized frequency list), average of the standardized frequencies of all words and word entropy.

##### Syntagmatic measures

Syntagmatic measures target information quantified at the level of a group of words that share a morphosyntactic, syntactic or semantic feature. These measures describe the behavior and distribution of these groups of words. Examples are the verb count, the first-to-third-person pronoun ratio, the burstiness (temporal behavior in terms of periodic, random and “bursty” recurrence) of passive voice markers, and the cosine distance between the average word vectors of two text segments.

### Calculation methods

This section describes how the values of the 351 linguistic measures in the 18 categories are computed. Lingualyzer includes (1) raw counts, (2) ratios, and (3) normalized counts. The simplest method is a (raw) count, which counts the total number of occurrences of a quantifiable unit, such as a word token. In the calculation of ratio scores, typically the count of one quantifiable unit is divided by the count of another. An example of a ratio is the number of nouns divided by the number of lexical items. A specific variant of the ratio scores are normalized counts or incidence scores. These scores divide the count of a quantifiable unit by the text length (i.e., number of word tokens in the text) to represent the density of a quantifiable unit. This is a score that is independent of the length of a text and allows for a comparison across texts. Because the resulting scores can get very small, we multiply them by 1000 to represent a count per 1000 words, a better readable representation commonly used in quantitative linguistic tools to represent normalized counts (Bengoetxea & Gonzales-Dios, 2021; Biber, 1988; Graesser et al. 2004). Incidence scores therefore always range between 0 and 1000. An example is the noun incidence score, i.e., the number of noun tags divided by the total number of words, multiplied by 1000. Raw counts, ratios and normalized counts are calculated for a large variety of descriptive, complexity and even linguistic distribution measures.

Even though the majority of the measures are calculated using one of those three methods, there still is a variety of measures that quantify aspects of the text through different methods. We specifically discuss the least familiar ones: Levenshtein distance, entropy, Zipf frequency and contextual diversity, Zipf’s law, burstiness and other dispersion measures, and cosine similarity.

#### Levenshtein distance

The Levenshtein distance denotes the minimal number of additions, deletions and replacements needed to transform one string into another (Levenshtein, 1966). There are multiple variants of this measure implemented. The Levenshtein character distance is a linguistic distribution measure that calculates the distance between different text segments in terms of character additions, deletions and replacements, while the Levenshtein word, lemma and PoS distance do the same, but for the words, lemmas and syntactic structure by looking at the word, lemma and PoS sequences of two text segments, respectively. The word-lemma Levenshtein distance, a linguistic complexity measure, uses the Levenshtein algorithm to denote the distance between each word and its lemma in a text segment through letter changes.

#### Entropy scores

Entropy scores denote the average information content of linguistic unit (Bentz et al., 2017; Gibson et al., 2019). We calculate the entropy for the words and characters in the text, using word unigrams and character unigrams respectively. These measures give an estimate of the predictability (and hence complexity) of the words and characters in a text segment.

#### Zipf frequency and contextual diversity

Since these measures are somewhat related and both based on the same external source (i.e., the word frequency databases), we discuss them together (Gimenes & New, 2016). The Zipf frequency of a word denotes the general or standardized frequency of usage (Van Heuven et al., 2014). The frequency is logarithmically scaled in order to be more readable, due to the large differences in frequency between frequent and infrequent words. Zipf frequencies are calculated by taking a logarithmic base of 10 from the unscaled frequency of occurrence for each word per billion words. Because we only include words that occur at least once per million words, the Zipf frequencies range between 3 and 9.^3^ We assign a Zipf frequency of 0 to words that do not occur in the frequency lists. Contextual diversity represents the percentage of all documents a word occurs in (Adelman et al., 2006). For both the Zipf frequency and contextual diversity, we included measures that calculate their word average in a text segment.

#### Zipf’s law

We also included information on the fit of Zipf’s power law to the word frequency distribution of a text segment (Zipf, 1949). The resulting fit is quantified by two values, namely (1) the estimated steepness of the slope of the distribution, and (2) the goodness-of-fit of the observed frequency distribution with the law, quantified through the *R*^2^ determination coefficient. These values tell us something about how word frequencies are distributed and how well that distribution adheres to the law. It has been argued that the steepness of the curve is negatively correlated with the number of cognitive resources available to the language user (Linders & Louwerse, 2023; Zipf, 1949).

#### Dispersion measures

Dispersion measures calculate how a group of words is distributed across a text segment. The burstiness measure indicates the temporal distribution of words based on their position in a text segment (Abney et al., 2018). Scores of +1 indicate “bursty” behavior, which means that words in a group tend to cluster together in smaller clusters, with long distances between these clusters. Scores of -1 indicate a more even distribution of the words across the text, i.e., the occurrence of words within this group is more periodic. Scores around 0 indicate random behavior. Because the original burstiness formula assumes a temporal sequence to be infinitely long (Abney et al., 2018), the formula does not approximate finite temporal sequences well into the “bursty” direction, especially for shorter sequences (Kim & Jo, 2016). Since texts by definition are finite sequences of words and can be arbitrarily short, we have therefore used the alternative formulation described by Kim and Jo (2016) that approximates finite and shorter sequences better.

Another measure used to assess the dispersion of a group of words is the average position in a text and its standard deviation. The average position is rescaled to a score between 0 and 1, with 0 denoting the start of the text segment and 1 the end. Finally, we have implemented a measure of dispersion that compares the number of occurrences in the first half of the text with the number of occurrences in the second half of the text segment. This ratio is scaled to represent a number between – 1, indicating all items occur in the first half of the text, + 1, indicating that all items occur in the second half of the text. 0 indicates the items are equally distributed between the two halves of the texts.

#### Word vectors

The word vectors can be used to calculate a semantic representation by taking the average vector over all words in the text segment. For each word, we retrieved the word vector and averaged the vectors of all words in a sentence to create a semantic representation of that sentence. If a vector is not available for a word, we approximated the word by taking its lemma. Those words for which neither the word nor the lemma is available, are ignored. We furthermore removed all words with an occurrence of more than 4000 times per million words on both the news and blogs word frequency lists from WordLex (Gimenes & New, 2016). This roughly corresponds to the 20 most frequent words in English, including words such as *and*, *to* and *the*, but the exact nature of extremely frequent words varies depending on the language, with only four words being removed in Telugu and Korean, but 29 in French. Removing high-frequency words, typically grammatical items, is a frequent procedure to optimize distributional semantic measures (Landauer et al., 2007). The average vectors are then used to calculate the semantic similarity between different text segments. This is done through calculating the cosine distance between two vectors. A score of 1 indicates perfect similarity, meaning that the contents of the two text segments is identical, while a distance of 0 indicates that the text segments are completely semantically distinct. Because these average vectors only look at content and do not take into account the size of a text segment, they can be used to compare text segments at different levels and of different lengths.

### General overview of measures

Our multidimensional setup with the analysis of different text levels, quantifying different units in the text, using a varied set of measures naturally leads to a large number of measures and an even larger number of values. To be precise, Lingualyzer computes 3118 different values for 351 different measures, spanning 18 categories of measures described above, at document, paragraph and sentence levels of analysis.^4^ These numbers are summarized by category in Table 3.

**Table 3:** Overview of the 351 measures and 3118 values, summarized by category

Information type	Language-specificity	Morphological		Lexical		Syntagmatic	
(Measures)	(Measures)	Measures	Values	Measures	Values	Measures	Values
Descriptive (89)	General (7)	1	9	4	36	2	6
	Linguistic (82)	0	0	0	0	82	738
Complexity (105)	General (10)	2	18	7	39	1	5
	Linguistic (95)	2	18	8	72	85	765
Distributional (157)	General (144)	0	0	16	128	128	1024
	Linguistic (13)	1	20	6	120	6	120
Total (351)	General: 161 Linguistic: 190	6	65	43	395	304	2658

From Table 3, one might conclude that there is a strong bias towards syntagmatic measures. This is especially true when looking at the number of linguistic descriptive and complexity measures. Due to Lingualyzer quantifying units primarily at the word level, there are only few measures at the morphological level. This is however compensated for by the syntagmatic measures, of which a large part capture morphosyntactic properties. These properties are expressed at the morphological level, but summarized by morphosyntactic feature and hence defined at the syntagmatic level. The overwhelming presence of syntagmatic measures is furthermore caused by the fact that for each PoS tag and for each morphosyntactic feature, there are multiple measures defined. For example, for burstiness and all other general distributional measures, there is a measure for each PoS tag and morphological feature, leading to a disproportionally large set of measures for this category.

It is important to note that, even though general measures rely on language-independent measures, they do use the tokenized and word segmented representations from Stanza for the quantification of words. While tokenization and word segmentation is a straightforward process in most languages, it is not in some, such as Chinese and Vietnamese, where word boundaries are not marked by whitespaces. Moreover, even though general distribution measures do not need to rely language-specific information in their calculation, they do rely on language-specific resources for the definition of the word groups. In other words, general measures still in essence quantify linguistic information in a text segment, demonstrating that strict demarcations between the 18 categories are difficult to make.

Measures from the different information type categories (i.e., descriptive, complexity, distributional) are not necessarily fully mutually exclusive. Measures from different categories might correlate, and measures from one category might also be informative for measures in another category. The same applies to the categorization of the quantified text unit (i.e., morphological, lexical, syntagmatic). The main goal of the categorization was not to create a theory-informed categorization, but to summarize and describe the variety in the different measures in an understandable way.

### Comparison with existing tools

With the 18 categories that Lingualyzer distinguishes, we can now better compare the tool to the other available tools presented earlier in this paper. This comparison is presented in Table 4. As the table shows, very few tools contain general distribution measures or measures at the paragraph level. Yet almost all existing tools contain general complexity and linguistic descriptive measures. This is not surprising since the quantification of different word groups in the dictionary-based tools is primarily on a linguistic descriptive level. Most tools also contain at least one general complexity measure, such as the type-token ratio.

**Table 4:** Overview of categories and text levels included in existing text analysis tools

	General			Linguistic			Text level		
Name	Descriptive	Complexity	Distribution	Descriptive	Complexity	Distribution	Document	Paragraph	Sentence
Diction				•			•		
Biber Tagger		•		•	•		•		
DIMAP-MCCA	•	•		•			•		
LIWC	•			•			•		
Coh-Metrix	•	•		•	•	•	•		
L2SCA		•			•		•		
ReaderBench	•	•		•	•	•	•		
T-Scan		•		•	•		•		
TAACO	•	•		•	•	•	•		
TAASSC		•		•	•		•		
I				•			•		
Profiling-UD	•	•		•	•		•		•
MultiAzterTest	•	•		•	•	•	•		
LATIC	•	•		•			•		
SentSpace	•	•		•	•				•
Lingualyzer	•	•	•	•	•	•	•	•	•

The text level (i.e., document, paragraph, sentence) has only been marked if the majority of the measures can be applied.

Lingualyzer differentiates itself from existing tools in a number of ways. First, it treats all 41 languages equally and uniformly, so that all measures and all dimensions can be analyzed in each language. Together with Profiling-UD, this is a significantly larger number of languages than is supported in any of the other tools. This uniformity entails that measures are comparable across languages and that different languages can be compared with each other. Lingualyzer can furthermore analyze several general distributional properties of texts, something that is not possible in any of the other tools. Consequently, Lingualyzer has the largest variety of measures, closely matched by Coh-Metrix, ReaderBench, TAACO and MultiAzterTest. Finally, Lingualyzer is the first quantitative text analysis tool that in addition to the document level can easily summarize all measures on a paragraph and sentence level as well.

Profiling-UD seems to be very similar to Lingualyzer, as it also supports multiple languages, is data-driven, is very accessible, contains a large variety of measures and can be applied to answer a large array of research questions. The NLP pipeline is furthermore trained on the same data, namely the UD treebank (Nivre et al., 2020), although Profiling-UD uses a slightly older NLP tool (UDPipe). Lingualyzer targets a larger range of dimensions (in addition to morphological and syntactic dimensions, also semantic dimensions). Most notably, Lingualyzer captures general distributional aspects and distributional semantic aspects of language, whereas it does not capture syntactic complexity and syntactic relations in as much detail as Profiling-UD. Lingualyzer furthermore targets multiple text levels and is trained on a slightly newer set of models and version of the UD treebanks.

There are however also some limitations in Lingualyzer when comparing tools. Firstly, Lingualyzer only performs a surface level syntactic analysis. For example, unlike Coh-Metrix or Profiling-UD, Lingualyzer does not construct a dependency parse tree for a deeper syntactic analysis. We excluded this analysis due to the heavy computation required for such an analysis and the generally lower quality of the dependency parse annotations. Furthermore, an in-depth lexical semantic analysis is not possible due to the absence of cross-linguistic databases. Hence, word-specific properties such as semantic categories of words and rating scores on polarity and concreteness are currently impossible to incorporate.

### Usage of Lingualyzer

Lingualyzer is a data-driven tool that analyzes texts in terms of general and linguistic contents and quantifies this contents into a large range of values at sentence, paragraph and document level. Because Lingualyzer is data-driven, it does not make any prior assumptions that are text-specific or language-specific. Hence, it can analyze any text, regardless of whether it is a large or small document and whether it consists of multiple paragraphs or sentences.

Because Lingualyzer is data-driven, it can be used for many different purposes, including register analysis, (author) profiling, readability assessment, as well as cross-linguistic analyses such as typology studies and text comparisons across languages. However, the large number of 351 measures, totaling 3118 values, might not be practical for all applications. We provide the user two ways to reduce the seemingly combinatorial explosion of measures. First, the user can select a reduced set of 33 values that cover most of the 18 categories, providing a comprehensive summary of the measures that will likely be most frequently used by the average user. This summary is the most basic version of Lingualyzer and is generally recommended for less experienced users and users new to Lingualyzer. An overview of these selected measures is given in Table 5. These measures only cover the document level, as well as one linguistic distribution measure, namely the cosine distance, which covers the paragraph–document, sentence–document, paragraph–paragraph, and sentence–sentence levels. For a full description of these (as well as all other) measures, we refer to the online documentation of the tool (https://lingualyzer.com). For users who prefer more flexibility in choosing the types of measures presented to them, but would like a comprehensive albeit not overwhelming overview, we provide the possibility to filter on the six categories, the three text levels themselves, as well as on the statistics used to summarize the values of the sentence and paragraph levels.

**Table 5:** Overview of the 33 measures in the reduced set

Name	Text level	Information type	Language-specificity	Quantified unit
Word count	Document	Descriptive	General	Lexical
Sentence count	Document	Descriptive	General	Syntagmatic
Noun incidence	Document	Descriptive	Linguistic	Syntagmatic
Lexical item incidence	Document	Descriptive	Linguistic	Syntagmatic
Pronoun incidence	Document	Descriptive	Linguistic	Syntagmatic
Grammatical item incidence	Document	Descriptive	Linguistic	Syntagmatic
Verb incidence	Document	Descriptive	Linguistic	Syntagmatic
Word length	Document	Complexity	General	Morphological
Sentence length	Document	Complexity	General	Lexical
Paragraph length	Document	Complexity	General	Syntagmatic
Type-token ratio	Document	Complexity	General	Lexical
Word entropy	Document	Complexity	General	Lexical
Zipf steepness of curve	Document	Complexity	General	Lexical
Zipf goodness of fit	Document	Complexity	General	Lexical
Frequent word incidence	Document	Complexity	Linguistic	Lexical
Infrequent word incidence	Document	Complexity	Linguistic	Lexical
Word types per lemma	Document	Complexity	Linguistic	Morphological
Lexical-grammatical item ratio	Document	Complexity	Linguistic	Syntagmatic
First-third-person pronoun ratio	Document	Complexity	Linguistic	Syntagmatic
Definite-indefinite word ratio	Document	Complexity	Linguistic	Syntagmatic
Present-past tense ratio	Document	Complexity	Linguistic	Syntagmatic
Hapax legomena burstiness	Document	Distribution	General	Lexical
Adjective burstiness	Document	Distribution	General	Syntagmatic
Proper noun burstiness	Document	Distribution	General	Syntagmatic
Personal pronoun burstiness	Document	Distribution	General	Syntagmatic
Hapax legomena avg position	Document	Distribution	General	Lexical
Adjective avg position	Document	Distribution	General	Syntagmatic
Proper noun avg position	Document	Distribution	General	Syntagmatic
Personal pronoun avg position	Document	Distribution	General	Syntagmatic
Cosine distance	Paragraph-Document	Distribution	Linguistic	Syntagmatic
Cosine distance	Sentence-Document	Distribution	Linguistic	Syntagmatic
Cosine distance	Paragraph-Paragraph	Distribution	Linguistic	Syntagmatic
Cosine distance	Sentence-Sentence	Distribution	Linguistic	Syntagmatic

#### Licensing

Lingualyzer is free to use for researchers in the scientific community. It is licensed under the Creative Commons Attribution-NonCommercial-NoDerivatives 4.0 International (CC BY-NC-ND 4.0). If you use Lingualyzer in your research, please cite the current paper.

The dependencies of Lingualyzer are the Stanza tool, the Universal Dependencies Treebank, the wordfreq tool and the fastText word vectors. Stanza and wordfreq are licensed under the Apache License 2.0, and the fastText word vectors are licensed under the Creative Commons Attribution-Share-Alike License 3.0. All Universal Dependencies Treebanks that are used, with three exceptions, are licensed under Creative Commons licenses with about a third not allowing any commercial use (Creative Commons Attribution-NonCommercial-ShareAlike).^5^ The Catalan, Polish, and Spanish treebanks are licensed under the GNU General Public License, Version 3.

#### How to use Lingualyzer

**Fig. 2:**
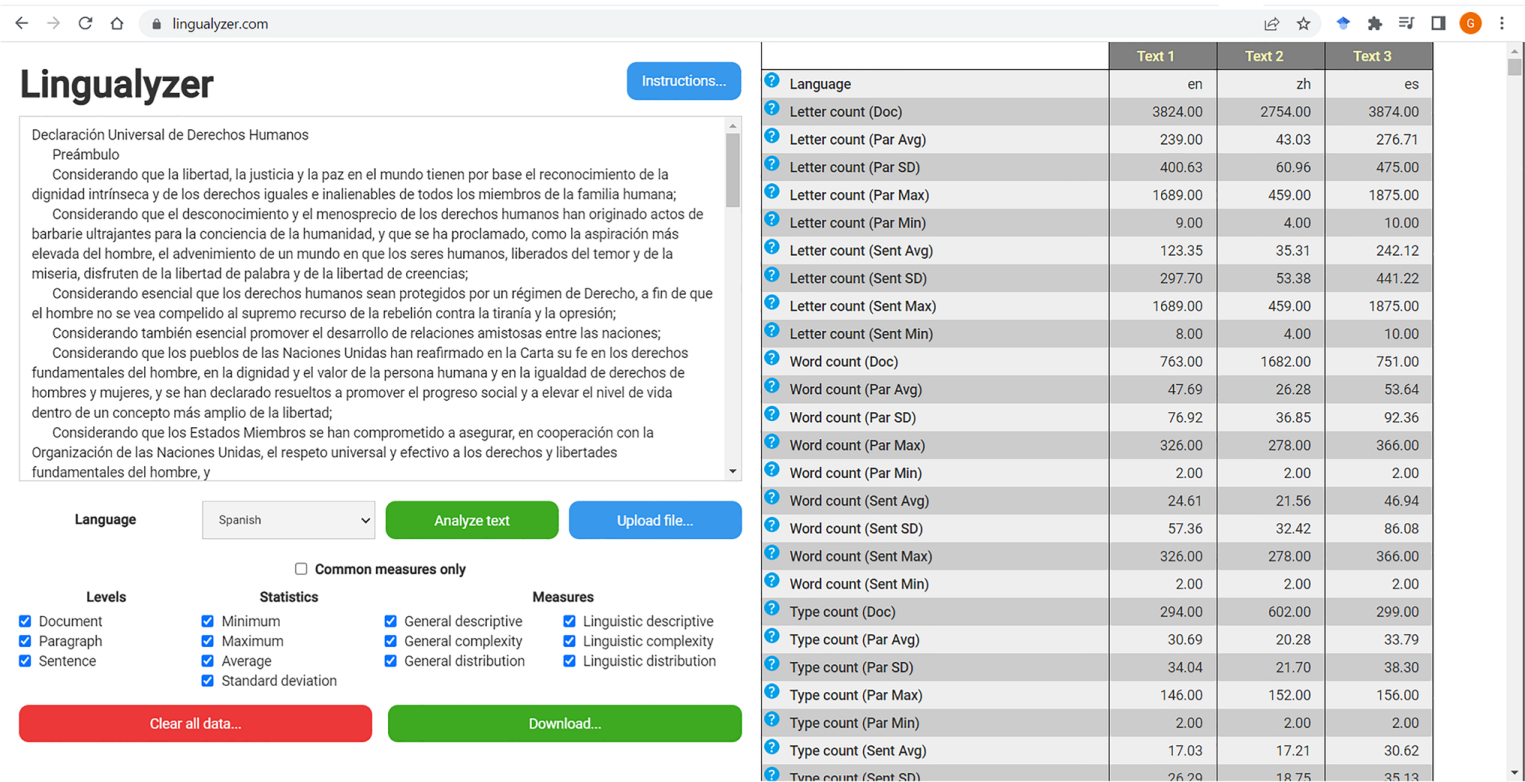
Illustration of the web interface of Lingualyzer

Lingualyzer is accessible for free through an online interface at https://lingualyzer.com/. The interface was developed in such a way that it is intuitive and easy to use, also for users new to Lingualyzer. An illustration of the interface is shown in Fig. 2. The user can either enter the text to be analyzed in a textbox, or can upload a text file consisting of the individual text to be analyzed. Uploaded texts must be submitted in text format. Users can enter texts that are up to approximately 40,000 characters long. Adhering to privacy concerns, Lingualyzer does not store or use any of the texts that are processed, nor does it store or use any of the processed output. User texts are deleted from the server when the analysis is completed.

Next, the user can select any filters that are needed to provide a (more) concise output of the Lingualyzer measures. Lingualyzer automatically selects the language for which the text needs to be investigated, but the user could also select the language manually prior to the analysis. The processing of text generally only takes a relatively small amount of time, typically in seconds the results are returned, though more time is needed for larger texts given the larger number of computations. Larger documents could take up to 2 min to analyze. Documents above the recommended limit might take a very long time to be completed by the tool. The Lingualyzer results are shown in a table consisting of a column with the title of the text, the language for which the text is analyzed, and the measures that have been selected by the user. For each additional text that is analyzed, an additional column is added to make a comparison of results across different texts straightforward. The results can be copied and pasted in a spreadsheet, but can also be downloaded in “.txt” format. The user is given the choice in downloading the full or just the filtered results.

#### Potential applications

The primary goal of Lingualyzer is to provide researchers with the possibility of analyzing texts across a large number of different languages. Lingualyzer supports 41 different languages from ten different language families, allowing researchers across a large and varied language landscape to perform quantitative linguistic text analyses. Many findings for English could potentially be validated across other languages and many new research questions can be investigated for new languages.

Exploring the possibilities of performing cross-linguistic analyses is a promising direction due to Lingualyzer computing the exact same measures across the languages supported. Moreover, the models were trained using the same algorithms with the underlying data based on a unified annotation framework. This means that the output of Lingualyzer is comparable across each individual language. While not all measures are meaningful when compared across languages, for example due to the absence of a morphological feature in some languages (e.g., definiteness not being marked in Chinese and Russian), the unified annotation framework of the UD Treebanks seem indeed to enable cross-linguistic comparisons with the general and linguistic complexity having been investigated across languages using the UD Treebank corpora and annotations (Bentz et al., 2023; Berdicevskis et al., 2018).

Lingualyzer captures a wide variety of different aspects in texts on different dimensions using a language-agnostic and text type-agnostic approach, and could therefore potentially be used in many “classic” quantitative text analysis applications, such as text and author characterization (Biber, 1988; Juola, 2008; Tausczik & Pennebaker, 2010), and readability and complexity assessment (Dascalu et al., 2013; McNamara et al., 2012). The relative simplicity of the measures (e.g., no complicated and error-prone computations, such as dependency parsing; cf. Qi et al., 2020) is likely an advantage as they might be more robust across different text types.

Lingualyzer furthermore provides summary statistics on a paragraph and sentence level and is unique in providing information consistently at three different text levels (i.e., document, paragraph, and sentence). These summarization statistics provide more localized information and could therefore potentially be very useful in for example (linguistic) stimuli creation, validation and analysis (Cruz Neri & Retelsdorf, 2022a; Dodell-Feder et al., 2011; Trevisan & García, 2019).

Finally, because of the large number of values computed by Lingualyzer, it can serve as feature input for computational algorithms. For example, such feature input can be used to train computational models that classify the truthfulness of (political) statements (Mihalcea & Strapparava, 2009; Rashkin et al., 2017), or detect humor and irony (Barbieri & Saggion, 2014; Reyes et al., 2012). Finally, the input can also be used to investigate if and how complex neural networks encode linguistic information with the goal of making such models insightful and explainable (Miaschi et al., 2020; Tuckute et al., 2022).

## Validation of Lingualyzer

Any computational linguistic tool ideally needs to be validated. McNamara et al. (2014, p. 165) distinguish intrinsic validation (testing that the tool does what it is supposed to) and extrinsic validation (evidence in terms of widespread use and acceptance by a community, for instance the discourse community).

Most tools given in Table 1 have been validated intrinsically. Most notably, Coh-Metrix was validated by comparing the output of texts with a high versus low cohesion, considering relative differences between the measures for the two conditions (McNamara et al., 2010). Coh-Metrix has furthermore been validated as a measure for differentiating several text characteristics, such as language registers (Louwerse et al., 2004) and authorship (McCarthy et al., 2006). Each version of LIWC was evaluated on a corpus with different text genres, where the consistency of word use within a dictionary category was measured across texts from different genres (Boyd et al., 2022). Apart from an evaluation by the authors themselves, LIWC has been incredibly popular and has been validated in numerous psychological domains (Tausczik & Pennebaker, 2010). MultiAzterTest was evaluated using yet another validation technique. The authors evaluated the correctness of the readability assessments made by their tool and compared them to the same assessments made by Coh-Metrix, taking the latter as a baseline (Bengoetxea & Gonzales-Dios, 2021).

These types of intrinsic validation are called *instrument validity*. The tool under investigation is validated for a particular purpose, for example readability assessment or register analysis. A prerequisite for *any* instrument validity, however, is to prove that the individual measures are both reliable and consistent. This is called *instrument reliability*. This is perhaps the most critical type of evaluation. Even though one may assume that the developers of an analysis tool have taken all care to make sure that the produced values are correct, instrument reliability is generally not reported. In fact, from all the tools in Table 1, we only know of two that have been validated using an instrument reliability study: L2SCA and LATIC. The syntactic annotations and the 14 measures in L2SCA, a tool for measuring the syntactic complexity in texts from non-native speakers of English, were verified by two annotators on a small subset of a corpus of English essays written by native Chinese speakers, demonstrating a high reliability of the tool both at the level of automatic annotations and the measures (Lu, 2010). The evaluation procedure of LATIC was very similar. Part-of-speech annotations in LATIC were manually verified in English and German through human annotations on a small sample from corpora containing fiction and news articles respectively (Cruz Neri et al., 2022b). Next, using five short texts, taken from introductory texts of questions from a science assessment, the measures were calculated by human annotators and correlations between the annotators and the output from LATIC were computed. No significant differences between the measures calculated by the annotators and the LATIC output were found.

Data-driven or hybrid tools with a large number of linguistic features have not reported instrument reliability, or such reports are at least not distributed through academic outlets. The reason for this is likely that computing the reliability is a very tedious and time-intensive process, due to the often many measures in these tools and the complex nature of the measures. It is therefore no wonder that the instrument reliability was investigated for tools with a relatively small number of easy-to-compute measures such as L2SCA and LATIC (cf. Table 1).

We provide both types of intrinsic validation: an instrument reliability study and an instrument validity study.

### Instrument reliability

#### Lingualyzer dependencies

In order to compute the output of the measures, Lingualyzer uses several computational linguistic resources. The reliability and validity of any computational tool is inherent to the quality of its external resources. The Stanza toolkit is used as an NLP pipeline to process (i.e., segment and annotate) the raw text and is thus used in all calculations. The word frequency and contextual diversity information from WorldLex are used in some measures to determine general use of words and fastText word vectors are used for comparing the semantic similarity across text segments.

For a reliable text quantification system it is important to have accurate annotations on all relevant tasks of the NLP pipeline. Stanza models have been intrinsically validated with each model having been individually evaluated on each NLP task. Stanza models typically have a high performance on all NLP tasks with average F1 scores above 85% on all the different tasks when looking at all the pre-trained models (Qi et al., 2020).

It is, however, difficult to assess the general robustness of Stanza and its applicability to different text registers and genres across languages, due to differences in genres and registers contained in the training data for each model, the size of the corpus and the quality of the annotations. Unfortunately, there are only few studies that investigated the annotations of Stanza for different registers and genres (Păiș et al., 2021; Sadvilkar & Neumann, 2020; Sirts & Peekman, 2020). Despite the fact that Stanza is a relatively new tool, it has already widely been used as a processing pipeline in many studies with texts from very different genres. For example, it has been used as a processing pipeline for detecting phishing e-mails (Gualberto et al., 2020), identifying comparative questions (Bondarenko et al., 2022), and investigating statistical tendencies in transcribed spoken dialog (Linders & Louwerse, 2023).

To ensure the highest possible reliability of the Stanza models in texts from different registers than trained on, we considered additional selection criteria for the inclusion of Stanza models and languages, and the use of Stanza annotations. First, we only included languages for which an accuracy of more than 80% was achieved on all relevant NLP tasks (i.e., tokenization, sentence segmentation, multi-word token expansion, lemmatization, PoS tagging and morphological feature annotation). Moreover, we only included models that were trained on at least 40,000 word tokens. Finally, if multiple models were available for a single language, we preferred the largest model or the model trained on the most varied corpus data in case there were only small differences in corpus size. Finally, we excluded measures based on annotations related to certain morphological features due to reliability concerns, such as abbreviations, mood and aspect which are also not consistently annotated across languages. For the same reason, we also did not use dependency parses, since they have a demonstrated lower performance (Qi et al., 2020).

For a small subset of the available languages, the word frequency and contextual diversity WorldLex databases were validated on a lexical decision task, showing significant correlations between reaction times of individual words and the frequency and contextual diversity (Gimenes & New, 2016), variables that have been hypothesized to strongly correlate in the psycholinguistic literature (Brysbaert & New, 2009). A subset of the fastText vectors were validated on a word analogy task in ten different languages (Grave et al., 2018), a common way to validate word vectors, given their rather abstract representation (Schnabel et al., 2015), though not without problems (Faruqui et al., 2016). The fastText vectors, albeit in most tasks not the best-performing word vector model (Wang et al., 2019), are widely used in many areas of natural language processing, owing to the unique availability of vectors in many different languages and the ability to represent unseen words (Lauriola et al., 2022).

#### Lingualyzer measures

In addition to a validation of its dependencies, the instrument reliability of Lingualyzer itself needs to be assessed. Here we report the instrument reliability for both English and Dutch, the two languages for which the authors who performed the manual verification had (near) native proficiency. Investigating all 41 languages is not necessary, because the implementation of the measures is independent of the selected language. The evaluation was done on the document level for the measures that analyze individual text segments, and on a sentence-sentence level for the measures that analyze and compare different text segments (i.e., linguistic distribution measures). A human validation of all 3118 values is also superfluous, due to the repetition of calculations and code at each of the text levels – yet we did check for any discrepancies across values. Values at the document level are, where possible, calculated using a bottom-up approach, combining the values from the lower levels. Thus, any calculation error at a lower level will cascade to the document level. For the linguistic distribution measures, the sentence–sentence level was chosen, due to the short texts used in the validation.

Manually computing the values on a single text large enough to cover all measures included in Lingualyzer is prone to human error in the human calculations, and it is difficult for peers to evaluate the results. We therefore opted for generating individual sentences that specifically target a measure. To avoid any biases on our end, we queried OpenAI’s ChatGPT (OpenAI, 2023), which we prompted for a sentence or short text with multiple instances of a characteristic specific to each measure.^6^ We performed the same sentence generation process for both English and Dutch. Some of these sentences were re-generated, adapted manually, or substituted with an earlier generated sentence in case ChatGPT did not yield a sentence that included instances of the quantified unit by the measure. Hence, for each of the 351 measures, we generated a single targeted sentence or short text on which the respective measure was evaluated. Generated texts consisted primarily of a single sentence each with approximately ten words, except for the sentences that required to be embedded in paragraphs which included 2–4 sentences, and for the cases where two sentences were needed, i.e., the linguistic distribution measures (see details below).

For all generated sentences, the Lingualyzer value was calculated by hand, *not* using Lingualyzer. External scripts were used for calculations that were infeasible to do by hand or if a specific resource (e.g., a word vector or word frequency) was needed. These values were then compared with the values generated by Lingualyzer and any discrepancies were investigated and resolved. We removed measures that yielded inconsistent annotations, such as measures based on negation markers, aspect, and mood. Due to the re-use of many of these annotations in different measures, this resulted in the removal of 84 measures. The removal of these measures guaranteed consistency within a language, but more importantly consistency across languages (i.e., a measure may have worked well for one language, but not for another), albeit with the sacrifice of a reduction of measures. Consequently, a perfect correlation was obtained between the Lingualyzer output and the human computations for all Lingualyzer measures for both Dutch and English. The dataset with the artificially generated sentences and the corresponding human-validated values can be viewed on the Lingualyzer website under the “Instructions”. These sentences can, in addition to being used for verifying Lingualyzer, also serve as examples with the aim of making the measures more insightful. The statistics of the English texts can be found in Table 6.^7^

**Table 6:** Statistics in number of words, word length, number of sentences and sentence length for Lingualyzer validation sentences

	Number of words	Word length	Number of sentences	Sentence length
Mean	11.25	5.21	1.17	9.69
SD	5.88	0.77	0.49	2.45
Min	1.00	3.00	1.00	1.00
Max	69.00	9.00	4.00	24.00

Sentence length is computed in number of words

**Table 7:** Percentage of Lingualyzer matching measures that give the correct value according to the human-validated gold-standard

Tool	Correctness (%)	*n*
Lingualyzer	100.00	351
Coh-Metrix	100.00	16
MAT	91.67	12
Profiling-UD	22.22	18
MultiAzterTest	97.37	38
LATIC	88.24	17

Sample size is determined by measures that overlap with Lingualyzer.

#### Comparison with existing tools

##### Human-validated sentences

Having established a perfect match between the Lingualyzer output and the human performance, we next compared these findings with a subset of existing tools, as reported in Table 1. Coh-Metrix (Graesser et al., 2004; McNamara et al., 2014), the re-implementation of the Biber Tagger, the Multi-dimensional Analysis Tagger or MAT (Nini, 2019), Profiling-UD (Brunato et al., 2020), MultiAzterTest (Bengoetxea & Gonzales-Dios, 2021) and LATIC (Cruz Neri et al., 2022b). These tools were chosen because (1) they were publicly available and (2) contained at least five measures that could be mapped onto a Lingualyzer measure. ReaderBench would also qualify for inclusion in the analysis, but was unfortunately unavailable at the time the analysis was conducted. We created a mapping from Lingualyzer measures to the measures in each of these tools. Where a match was less apparent, we made adjustments. These concern the following. First, MAT, Profiling-UD and LATIC use incidence scores to represent the occurrence per 100 words, while all other tools represent the same scores per 1000 words. Hence we multiplied the incidence scores of Profiling-UD and LATIC with a factor 10 to match those in Lingualyzer. Second, some values in Lingualyzer were represented by two individual values in other tools. For example, Coh-Metrix contains incidence scores for both first-person singular and first-person plural pronouns separately, while Lingualyzer only contains a single incidence score for both singular and plural first-person pronouns. In these cases, we added up the scores of these individual values.

We made a distinction between measures where an exact correspondence was expected and measures where an approximate correspondence is to be expected. Approximate correspondences were expected when NLP processing algorithms were trained on different datasets with different tagsets, leading to slight differences the resulting scores. Moreover, some measures that represent the same information were calculated slightly differently. One example is the type-token ratio over the first 100 words in Profiling-UD, which can only approximate the more holistic moving average type-token ratio in Lingualyzer that is calculated over all possible windows of 100 words in a text segment. Another example is the calculation of the cosine distance, which in Coh-Metrix is based on latent semantic analysis, while it is based on average word vectors in Lingualyzer.

Because only one of the tools (Profiling-UD) included the Dutch language, contrary to our previous instrument reliability assessment, we only compared the output of the tools on the English sentences in this analysis. Of the 351 measures in Lingualyzer, there were 56 measures that had an equivalent in one or multiple existing tools. MAT had the smallest number of equivalent measures with 12, while MultiAzterTest had the largest with 38.

For each tool, we calculated the percentage of the measures that returned the correct value, based on the human-validated gold standard (See 3.1.2 Lingualyzer measures). Here we mitigated possible effects of different rounding strategies by allowing for a very small margin of error. The correctness percentages are summarized in Table 7. The performance of Lingualyzer for the 351 sentences equals human performance. All other tools only made minor mistakes when compared with the manually computed output (and hence the Lingualyzer output), resulting in correctness percentages between 88 and 100%, supporting their general reliability. The only notable exception is Profiling-UD, which yielded a low correctness percentage of 22%. This, however, can almost exclusively be traced back to the fact that punctuation marks are seen and counted as individual word tokens and that consequently all measures that rely on this count, such as all incidence scores and word length, return an incorrect value. Note, however, that a meaningful comparison of the correctness percentages across tools is not possible due to differences in the exact nature of the measures and the number of measures that overlap with Lingualyzer.

Just like the sentences used for the validation of Lingualyzer measures (3.1.2), the dataset with the mapping of the Lingualyzer measures to the measures of the tools used in the comparison can be found on the Lingualyzer website under “Instructions”.

##### Actual texts

The instrument reliability analysis using short sentences that targeted individual measures is welcome, as it (1) allows to verify the accuracy of measures, and (2) provides examples of the measures to the user. However, one may argue that such an analysis does not represent a naturalistic scenario in which Lingualyzer would be used. The results from this evaluation can therefore only be interpreted as validating that the measures reliably calculate the correct value.

To evaluate Lingualyzer with naturalistic data we compared the output of the Lingualyzer measures with the same tools as in the previous analysis on texts that likely more closely represent actual use cases. These five tools are available in English. The next most common language among the tools in Table 1 is Spanish. Three out of these five tools support Spanish, which is why we included it in this analysis as well. Moreover, we investigated three different texts from three different genres: a fiction book, a very recent news article and a transcript from a free-flow spoken dialog between two participants. From Project Gutenberg, we retrieved the first chapters of the following fiction books in English and Spanish, respectively: “Alice's Adventures in Wonderland” and “El idilio de un enfermo”.^8^ We selected the following news articles: “Diana knew she wouldn’t be queen — and doubted Charles wanted the crown” from The Washington Post and “El misterioso asesinato de Guillermo Castillo, el chef del pueblo” from El Mundo.^9^ Finally, for the spoken dialog transcripts, we selected the dialog with id “sw_0243_3513” from the Switchboard Dialog Act Corpus (Stolcke et al., 2000), and from the Spanish CallFriend corpus, we selected the dialog with id “4057” (MacWhinney, 2007). Texts were converted to a “.txt” format and encoded using UTF-8. In addition, all newline characters that solely served to enhance readability were removed to ensure homogeneity in formatting. For the spoken conversations, annotations were removed, and each turn was separated into a single paragraph.

Given the nature of the values, we computed a non-parametric Spearman rank-order correlation for each text and tool, correlating the values for each tool with the corresponding Lingualyzer values. These results are shown in Table 8. Note that only 42 different values at most were compared across the tools, only a small subset of all values computed in Lingualyzer, and that a comparison across tools is again not possible, due to differences in measures that are correlated. Note further that in this analysis, next to the measures with an exact correspondence to a Lingualyzer measure, we also included measures with an approximate correspondence. Overall, correlations are very high with most correlations *r* > .95, showing consistency in the measures across the tools, across languages and across genres with the exception of dialog. Even though the sample size is small, with only one text per language and text genre, it is clear that correlations are the lowest for dialog and especially low with MAT in English and LATIC in Spanish. In sum, this highlights that, despite the differences in annotations and possible mistakes, tools are comparable to Lingualyzer on the small subset of overlapping measures, on fiction and news articles and to a lesser extent on spoken dialog transcripts.

**Table 8:** Spearman’s rank correlation between Lingualyzer and the respective tool for each language and text pair

	Fiction		News		Dialog		*n*	
	English	Spanish	English	Spanish	English	Spanish	English	Spanish
Coh-Metrix	.982		.976		.893		25	
MAT	.989		.940		.669^†^		13	
Profiling-UD	.984	.995	.989	.961	.986	.934	19	18
MultiAzterTest	.996	.995	.988	.956	.989	.981	42	40
LATIC	.971	.978	.953	.978	.978	.652^†^	17	14

All correlations are significant at *p* < .005, unless otherwise stated. ^†^ *p* < .05.

### Instrument validity

In addition to the instrument reliability – comparing the outcome of Lingualyzer measures with those by human raters and existing tools – instrument validity is relevant. One of the primary aims of Lingualyzer is to open up the possibilities for researchers in the behavioral science community to study one or multiple languages beyond English. To facilitate this, we aimed to make all measures available in all languages and make each measure as comparable as possible across languages. However, in order for Lingualyzer to be a reliable tool to analyze or compare multiple language, differences in output across languages need to be systematic. We selected a parallel corpus, the translations of the Universal Declaration of Human Rights (UDHR).^10^ Because the contents of the document is supposedly identical across translations, we expected the differences in output to be caused by linguistic differences between languages. We predicted that linguistic differences – and therefore the “linguistic distances” were smaller for more closely-related languages (Chiswick & Miller, 2005; Wichmann et al., 2010). We correlated the linguistic differences extrapolated from a bottom-up approach (i.e., the Lingualyzer output from the UDHR translations) with linguistic differences extrapolated from a database of language typology, which we will call a top-down approach.

The fundamental difference between a bottom-up and top-down approach to comparing language is that a bottom-up approach relies on actual corpus data and the statistical patterns that can be found in this data, and the top-down approach relies on descriptions of generalized patterns in language by expert judgments. In the field of language typology, the two approaches are referred to as token-based and type-based typology, respectively (Levshina, 2019). A bottom-up approach has been used to show and explain the universality of several quantitative or statistical linguistic laws (Bentz & Ferrer-i-Cancho, 2016; Bentz et al., 2017; Piantadosi et al., 2011), while a top-down approach has been used to explain how languages are different from and related to each other (Bickel, 2007; Comrie, 1989; Georgi et al., 2010).

For the bottom-up approach, the UDHR corpus was chosen as our data source because the information is formal and leaves little room for ambiguity. It is therefore less susceptible to differences in meaning or content across different translations, for instance due to stylistic differences or figurative language use. The UDHR corpus is rather small, consisting of only roughly 63 paragraphs and 86 sentences. We removed all metadata and used Lingualyzer to compute the results for all 351 measures (and the resulting 3118 values) for all translations in each of the 41 languages. A comparison of similarities and differences in the output, quantified through a linguistic distance calculation would then allow for identifying how similar the languages are.

But how do we quantify this linguistic distance between languages? Due to the widely varying scales of the values of the Lingualyzer output, simply computing a Euclidean distance would bias the distance towards the measures with the larger scale. We therefore normalized the data by computing *z* scores for each of the values. The advantage of this normalization is that the resulting values are not only centered around 0, but are also comparable in their deviation from the mean across languages. We then removed values that had a perfect correlation with another value, when looking at the values across languages, since these values are redundant and therefore uninformative. Similarly, we removed the measures where all values were the same across languages. Finally, we computed the Euclidean distance between the *z* scores of the different values for each language pair.

For the top-down approach, we used the World Atlas of Language Structures (WALS) to extract typological features for all languages available in Lingualyzer (Dryer & Haspelmath, 2013). The current version of WALS has 192 different features which each can take between two and 28 values. Defining distances between languages, based on the typological features is not straightforward. Here we closely followed Rama and Kolachina (2012), who created a binary feature matrix from the typological features, which was subsequently used to quantify the distances between languages. Unfortunately, not every feature is defined for all languages, leaving many feature values undefined. Therefore, similar to Rama and Kolachina (2012), we removed features that were not shared by at least 10% of the languages, to avoid creating feature vectors that are too sparse. We then converted each feature with *k* different values into *k* different binary features, marking the presence or absence of that particular feature in a language, similar to Georgi et al. (2010), and Rama and Kolachina, (2012). Since Serbian and Croatian are combined in WALS, we used the same binary feature vector for both languages. In total we had 515 binary features, covering 66.1% of all possible values across languages. Afrikaans and Slovak had to be removed from further analysis, because these languages contained too few features, resulting in language pairs with no overlapping features. This meant that we were unable to define distances between these language pairs. For the remaining language pairs, we quantified the distances between language pairs using the Hamming distance, which is the normalized count of the number of values that are equal in the two vectors. Here, all feature values that were undefined for one or both of the languages, were removed prior to the calculation of the distance.

**Fig. 3:**
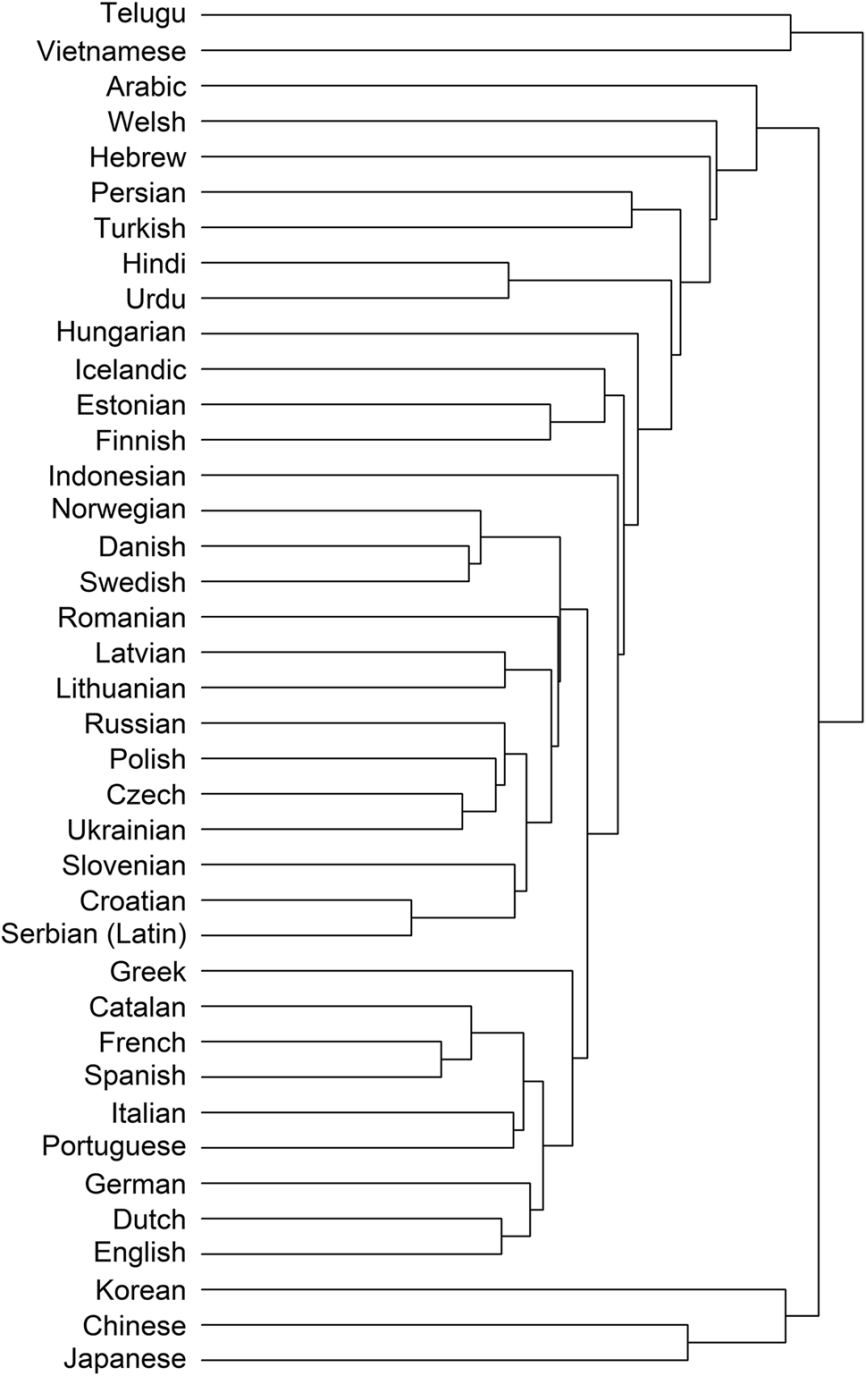
Dendrogram created from a distance matrix based on differences in Lingualyzer output between languages

**Fig. 4:**
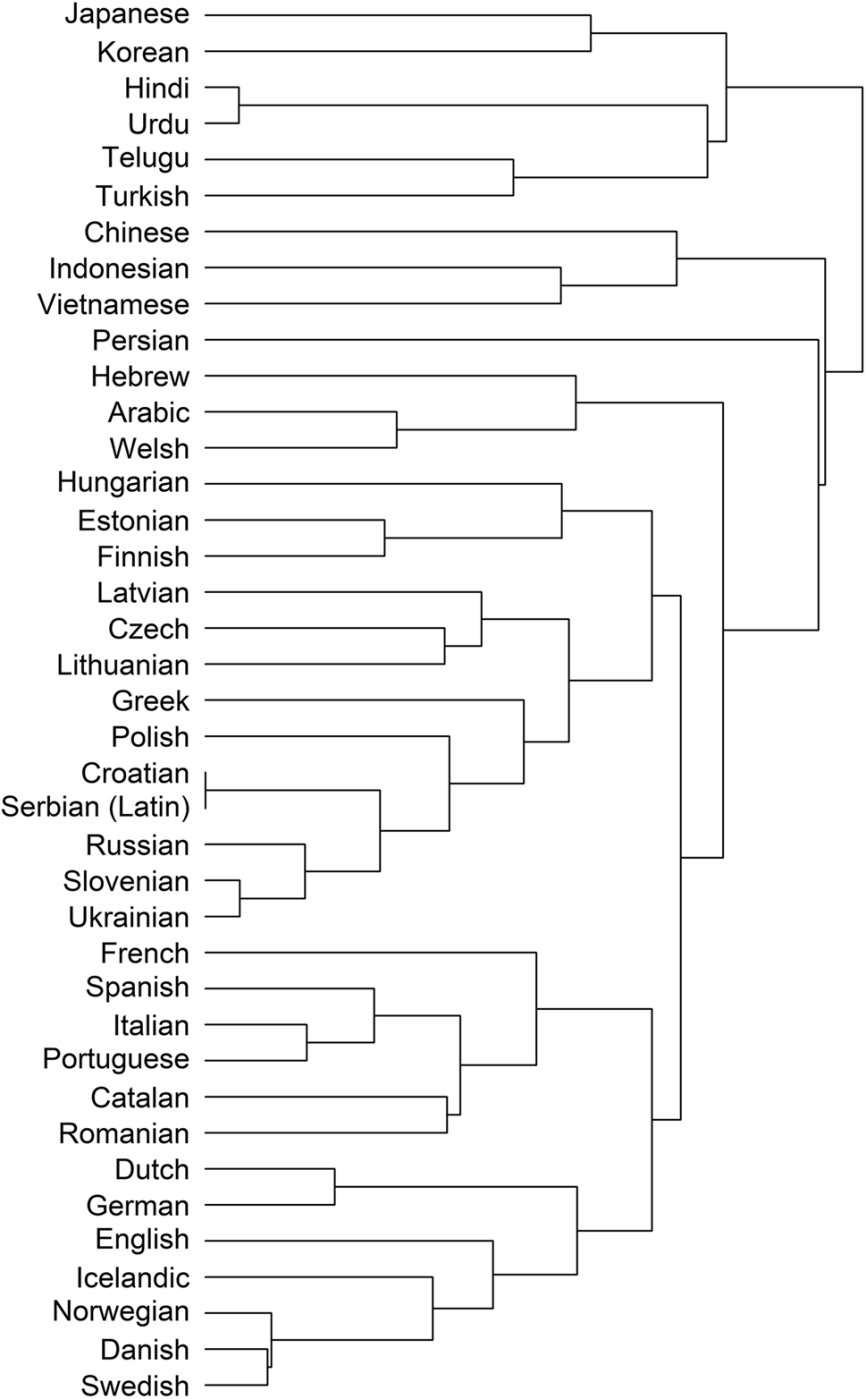
Dendrogram created from a distance matrix based on typological differences between languages

We then performed a hierarchical clustering. In Figs. 3 and 4 we summarized the hierarchical clusters into dendrograms for the Lingualyzer (Fig. 3) and typological distances (Fig. 4). The similarities between the Lingualyzer and the language typology dendrograms are illustrative but obvious. In both dendrograms, languages from the same family or branch tend to cluster together. Note that the Lingualyzer clustering cannot be explained by the language script. For instance, Hindi and Urdu, two languages that are similar yet do not share the same script, cluster together.

In order to compare the similarity between the hierarchical clusters, we computed the cophenetic correlation coefficient, a technique that allows for comparing the similarity of clusters created through hierarchical clustering, i.e., dendrograms (Sokal & Rohlf, 1962). Similar to an “ordinary” correlation, its values range between – 1 and + 1, indicating the strength of a negative or positive correlation. The correlations between the hierarchical clusters created from the Lingualyzer distances and typological distances are shown in Table 9. Here, we report not only the results where all Lingualyzer measures were used in calculating the distances between language pairs, but also where we used subsets of the Lingualyzer measures. These subsets include measures from the following categories: document level, general descriptive, linguistic descriptive, general complexity, linguistic complexity, general distribution and linguistic distribution. Despite the very different approaches to establishing the distances between language pairs (i.e., a bottom-up, token-based, data-driven approach versus a top-down, type-based, expert judgement-based approach), a moderate to strong correlation between the resulting clusters of both approaches is found, showing that a significant portion of the variance of the distances between languages on a parallel corpus, calculated using the Lingualyzer measures, can be attributed to typological or linguistic differences between languages.

**Table 9:** Cophenetic correlation coefficients between Lingualyzer and WALS for different subsets of Lingualyzer measures

Lingualyzer measures subset	Cophenetic correlation
All	.690
Document (no linguistic distribution)	.664
General descriptive	.433
Linguistic descriptive	.699
General complexity	.491
Linguistic complexity	.642
General distribution	.716
Linguistic distribution	.433

Zooming in on the different subsets of Lingualyzer measures, we can observe a slightly lower correlation for the document measures, compared to all other measures. The linguistic descriptive and complexity measures result in a significantly higher correlation than their general counterparts. This is not surprising, given that the WALS features are linguistic by definition. For the same reason, the general distribution measures result in a similar correlation, as they quantify the distribution of primarily linguistic variables and are typically language-specific. Because the linguistic distribution measures quantify similarities between different text segments, they are less indicative of variation between languages. Still, we find a low-to-moderate correlation for this subset.

These results, albeit only illustrative for a language typology study, demonstrate an example of instrument validity paving the way for the use of Lingualyzer in cross-linguistic studies and comparisons. The moderate-to-strong correlations indicate systematicity in the variation across languages and thus also consistency in the measures across the languages. This is an important prerequisite for any analysis involving multiple languages. Finally, this validation study also demonstrates one potential use case of Lingualyzer, namely investigating cross-linguistic generalizations. One exciting potential extension of this study is to investigate whether the output of Lingualyzer can predict the presence or absence of a typological feature in a language, based on just usage-based language data.

## Discussion and conclusion

This paper presented Lingualyzer, an easy-to-use multilingual computational linguistic tool that can be used for text analysis across a multitude of features. Lingualyzer analyzes text on 351 measures, categorized into 18 categories, resulting in 3118 values across 41 languages. Compared with other computational linguistic tools available, Lingualyzer is unique because it allows for such a large number of different languages on such a large number of computational measures, with measures that are available and comparable in all languages.

As with every tool, Lingualyzer has some limitations. First and foremost, Lingualyzer does not yet support batch processing. Each document has to be entered individually. This may not be practical when a large number of documents need to be processed, but to save resources and to avoid misuse of the tool, this is for now the most feasible option. Internally (i.e., not through the public web interface), Lingualyzer does allow for batch processing. Moreover, we are evaluating options to also allow batch processing for a larger audience. Second, the number of features that Lingualyzer uses to analyze text at different levels is large, but could be larger. However, because Lingualyzer allows for cross-linguistic analyses, consistency across languages is more critical than obtaining a maximum number of features. Conversely, we have taken care of not overwhelming the user with a magnitude of features providing a common-features-only option. Finally, Lingualyzer only covers less than 1% of the living languages in the world today. That is the disappointing news. However, the 41 languages Lingualyzer does cover, are the languages most commonly used. As with the measures that Lingualyzer includes, the languages it covers are based on a selection that ensures consistency in cross-linguistic analyses.

Even though most computational linguistic tools are presumably validated intrinsically – removing any bugs or inconsistencies – instrument reliability often tends not to be reported. For Lingualyzer, we have provided a few examples of instrument reliability: comparing the results of Lingualyzer with human performance (for two languages), and comparing its results with those of other tools. Evaluating the instrument reliability of a computational linguistic tool such as Lingualyzer is an immense task, which is virtually impossible to do across all languages and across all measures and values. Individual measures were validated on a representative set of sentences and (where applicable) compared to similar measures in existing tools across different genres. The validations reported in the current paper demonstrate that Lingualyzer measures are reliable. It is, however, important to stress that measures were not validated across all 41 languages. However, the potential for errors in other languages not included in the validation, has been minimized, first because errors for one language must apply to multiple languages and those errors have been removed for Dutch and English. Second, a careful pass through the selection of the measures (e.g., by not considering more error-prone annotations such as dependency parses) has furthermore minimized the chance of errors. Similar to it not being feasible to validate all 41 languages, not all 3118 values were individually considered. Here, too, errors that were to occur at one level must propel to other levels. Careful investigation at the sentence and paragraph level must have minimized (and as far as we can tell eliminated) errors at the other levels.

In addition to reporting the instrumental reliability, we also reported the instrumental validity of Lingualyzer by comparing its cross-linguistic output with that of a language typology. While the similarities between the hierarchical relationships from the Lingualyzer output and those from the language typology are obvious, some considerations are in place. First, the typological differences contain many missing binary values, resulting in the distance of each language pair being based on different typological features. So while the results are interesting and the Lingualyzer and typological dendrograms are comparable, our baseline, the typological distances, is at best an approximation. Moreover, the visualization through dendrograms purely illustrates similarities between languages, which do not necessarily correlate with genealogical relationships between languages. For example, while Welsh is an Indo-European language, in both dendrograms, it is close to the Afro-Asiatic languages Arabic and Hebrew, possibly due to these languages sharing some unique grammatical features, such as the widespread use of infixes. Similarly, while Romanian is overall typologically very similar to the other Romance languages, the isolation of Romanian compared with the other Romance languages, might have led to significant differences that are more apparent in the Lingualyzer measures than in the language typology. However, the analysis presented here is illustrative and should not be used as a full typology study. Yet, it does provide some useful insights in similarities and differences across languages.

We hope that with the availability of Lingualyzer, the behavioral science community has a useful computational linguistic tool to its availability. Many areas within the behavioral sciences and related fields do not necessarily have the computational linguistic expertise or programming skills to extract linguistic features from texts. Lingualyzer aims to fill this gap by providing behavioral scientists, including linguists, psycholinguists, corpus linguists, literary scholars, anthropologists, sociologists, and economists, with the opportunity to easily analyze text across different levels and a multitude of different dimensions. Because of its scope Lingualyzer can be used for a variety of purposes. But most importantly, Lingualyzer extends research often limited to languages spoken by a WEIRD community, and more specifically English language community, to languages spoken by a far larger community. We specifically hope that Lingualyzer allows for novel and innovative research in the behavioral sciences, pushes the boundaries of findings obtained for one language to 40 others languages, and offers explorations on similarities and differences across those languages.

## Data Availability

Lingualyzer can be accessed at the following webpage: https://lingualyzer.com/. The data used in the evaluation can also be found on this website. All other data sources, including the ones Lingualyzer uses, are reported in this article and are free to use for scientific purposes.

## Footnotes

1 Chinese and Japanese do not use whitespaces at all, while Vietnamese marks syllable boundaries, instead of word boundaries, with white spaces.

2 Note that language-independent measures still require a language-specific word segmentation algorithm to separate the raw text into countable word tokens. Language-independent measures, however, do not rely on linguistically informed annotations on these word tokens.

3 The theoretical upper bound is that a word occurs a billion times per billion words. In this case the Zipf frequency would be 9. However, typically, there are no words in languages with a Zipf frequency of 8 or higher, which would mean that a word occurs at least 100 million times per billion words.

4 It should be noted however that, despite the fact that we have defined 18 categories, the total number of categories for which we have implemented measures is 15, as there are three categories for which no measures are implemented. The reason is not that no such measures exist, but that these measures cannot be reliably implemented across languages.

5 See: https://stanfordnlp.github.io/stanza/available_models.html for more details about the licenses.

6 ChatGPT cannot be used for computing the outcome of the measures and can only help with generating sentences, as for high precision tasks it frequently yields erroneous results.

7 Note that the statistics for the Dutch sentences and paragraphs were comparable with those for English: for number of words (M = 11.35, SD = 9.14, range 1–141), word length (M = 5.41, SD = .98, range 3–11), number of sentences (M = 1.13, SD = .70, range 1–10), and sentence length (M = 10.07, SD = 2.41, range 1–21).

8 *Alice’s Adventures in Wonderland* can be found here: https://www.gutenberg.org/ebooks/11, and *El idilio de un enfermo* can be found here: https://www.gutenberg.org/ebooks/25777.

9 *Diana knew she wouldn’t be queen — and doubted Charles wanted the crown* can be found here: https://www.washingtonpost.com/history/2023/05/06/diana-coronation-king-charles-queen/, and *El misterioso asesinato de Guillermo Castillo, el chef del pueblo* can be found here: https://www.elmundo.es/espana/2023/05/05/6453e904e4d4d8c94a8b4584.html.

10 The translations can be found here: http://www.unicode.org/udhr/.

## References

[CR1] Abney, D. H., Dale, R., Louwerse, M. M., & Kello, C. T. (2018). The bursts and lulls of multimodal interaction: Temporal distributions of behavior reveal differences between verbal and non-verbal communication. *Cognitive Science, 42*(4), 1297–1316. 10.1111/cogs.12612 29630740 10.1111/cogs.12612

[CR2] Adelman, J. S., Brown, G. D., & Quesada, J. F. (2006). Contextual diversity, not word frequency, determines word-naming and lexical decision times. *Psychological Science, 17*(9), 814–823. 10.1111/j.1467-9280.2006.01787.x 16984300 10.1111/j.1467-9280.2006.01787.x

[CR3] Alvero, A., Giebel, S., Gebre-Medhin, B., Antonio, A. L., Stevens, M. L., & Domingue, B. W. (2021). Essay content and style are strongly related to household income and SAT scores: Evidence from 60,000 undergraduate applications. *Science*. *Advances, 7*(42). 10.1126/sciadv.abi9031 10.1126/sciadv.abi9031 PMC8514086 34644119

[CR4] Artetxe, M., Aldabe, I., Agerri, R., Perez-De-Viñaspre, O., & Soroa, A. (2022). Does corpus quality really matter for low-resource languages? In Y. Goldberg, Z. Kozareva, & Y. Zhang, *Proceedings of the 2022 Conference on Empirical Methods in Natural Language Processing* (pp. 7383–7390). Association for Computational Linguistics.

[CR5] Barbieri, F., & Saggion, H. (2014). Automatic detection of irony and humour in Twitter. In S. Colton, D. Ventura, N. Lavrac, & M. Cook, *Proceedings of the Fifth International Conference on Computational Creativity* (pp. 155–162). Association for Computational Creativity.

[CR6] Bender, E. M. (2009). Linguistically naïve!= language independent: Why NLP needs linguistic typology. In T. Baldwin, & V. Kordoni, *Proceedings of the EACL 2009 Workshop on the Interaction between Linguistics and Computational Linguistics: Virtuous, Vicious or Vacuous?* (pp. 26–32). Association for Computational Linguistics.

[CR7] Bengoetxea, K., & Gonzalez-Dios, I. (2021). MultiAzterTest: A multilingual analyzer on multiple levels of language for readability assessment. *arXiv preprint arXiv:2109.04870*. 10.48550/arXiv.2109.04870

[CR8] Bentz, C., & Ferrer-i-Cancho, R. (2016). Zipf's law of abbreviation as a language universal. In C. Bentz, G. Jäger, & I. Yanovich, *Proceedings of the Leiden Workshop on Capturing Phylogenetic Algorithms for Linguistics* (pp. 1–4). University of Tübingen. 10.15496/publikation-10057

[CR9] Bentz, C., Alikaniotis, D., Cysouw, M., & Ferrer-i-Cancho, R. (2017). The entropy of words—Learnability and expressivity across more than 1000 languages. *Entropy, 19*(6), 275. 10.3390/e19060275 10.3390/e19060275

[CR10] Bentz, C., Gutierrez-Vasques, X., Sozinova, O., & Samardžić, T. (2023). Complexity trade-offs and equi-complexity in natural languages: A meta-analysis. *Linguistics Vanguard, 9*(s1), 9–25. 10.1515/lingvan-2021-0054 37275745 10.1515/lingvan-2021-0054 PMC10234276

[CR11] Berdicevskis, A., Çöltekin, Ç., Ehret, K., von Prince, K., Ross, D., Thompson, B., Yan, C., Demberg, V., Lupyan, G., Rama, T., & Bentz, C. (2018). Using Universal Dependencies in cross-linguistic complexity research. In M.-C. de Marneffe, T. Lynn, & S. Schuster, *Proceedings of the Second Workshop on Universal Dependencies (UDW 2018)* (pp. 8–17). Association for Computational Linguistics. 10.18653/v1/W18-6002

[CR12] Biber, D. (1988). *Variation across speech and writing*. *Cambridge University Press.*10.1017/CBO9780511621024

[CR13] Bickel, B. (2007). Typology in the 21st century: Major current developments. *Linguistic Typology, 11*(1), 239–251. 10.1515/LINGTY.2007.018 10.1515/LINGTY.2007.018

[CR14] Blasi, D. E., Henrich, J., Adamou, E., Kemmerer, D., & Majid, A. (2022). Over-reliance on English hinders cognitive science. *Trends in Cognitive Sciences, 26*(12), 1153–1170. 10.1016/j.tics.2022.09.015 36253221 10.1016/j.tics.2022.09.015

[CR15] Bondarenko, A., Ajjour, Y., Dittmar, V., Homann, N., Braslavski, P., & Hagen, M. (2022). Towards understanding and answering comparative questions. In K. Selcuk Candan, H. Liu, L. Akoglu, X. L. Dong, & J. Tang, *Proceedings of the Fifteenth ACM International Conference on Web Search and Data Mining.* Association for Computing Machinery. 10.1145/3488560.3498534

[CR16] Boyd, R. L., Ashokkumar, A., Seraj, S., & Pennebaker, J. W. (2022). *The development and psychometric properties of LIWC-22.* University of Texas at Austin.

[CR17] Brunato, D., Cimino, A., Dell’Orletta, F., Venturi, G., & Montemagni, S. (2020). Profiling-UD: A tool for linguistic profiling of texts. In N. Calzolari, F. Béchet, P. Blache, K. Choukri, C. Cieri, T. Declerck, S. Goggi, H. Isahara, B. Maegaard, J. Mariani, H. Mazo, A. Moreno, J. Odijk, & S. Piperidis, *Proceedings of the 12th Language Resources and Evaluation Conference (LREC'20)* (pp. 7145–7151). European Language Resources Association.

[CR18] Brysbaert, M., & New, B. (2009). Moving beyond Kučera and Francis: A critical evaluation of current word frequency norms and the introduction of a new and improved word frequency measure for American English. *Behavior Research Methods, 41*(4), 977–990. 10.3758/BRM.41.4.977 19897807 10.3758/BRM.41.4.977

[CR19] Chiswick, B. R., & Miller, P. W. (2005). Linguistic distance: A quantitative measure of the distance between English and other languages. *Journal of Multilingual and Multicultural Development, 26*(1), 1–11. 10.1080/14790710508668395 10.1080/14790710508668395

[CR20] Comrie, B. (1989). *Language universals and linguistic typology: Syntax and morphology.* University of Chicago Press.

[CR21] Conneau, A., Khandelwal, K., Goyal, N., Chaudhary, V., Wenzek, G., Guzmán, F., Grave, E., Ott, M., Zettlemoyer, L., & Stoyanov, V. (2020). Unsupervised cross-lingual representation learning at scale. In D. Jurafsky, J. Chai, N. Schluter, & J. Tetreault, *Proceedings of the 58th Annual Meeting of the Association for Computational Linguistics* (pp. 8440–8451). Association for Computational Linguistics. 10.18653/v1/2020.acl-main.747

[CR22] Crossley, S. A., Kyle, K., & Dascalu, M. (2019). The tool for the automatic analysis of cohesion 2.0: Integrating semantic similarity and text overlap. *Behavior Research Methods*, 14–27. 10.3758/s13428-018-1142-4 10.3758/s13428-018-1142-4 30298264

[CR23] Crossley, S. A., Kyle, K., & McNamara, D. S. (2016). The tool for the automatic analysis of text cohesion (TAACO): Automatic assessment of local, global, and text cohesion. *Behavior Research Methods, 48*(4), 1227–1237. 10.3758/s13428-015-0651-7 10.3758/s13428-015-0651-7 26416138

[CR24] Crossley, S. A., Kyle, K., & McNamara, D. S. (2017). Sentiment analysis and social cognition engine (SEANCE): An automatic tool for sentiment, social cognition, and social-order analysis. *Behavior Research Methods, 49*(3), 803–821. 10.3758/s13428-016-0743-z 10.3758/s13428-016-0743-z 27193159

[CR25] Cruz Neri, N., & Retelsdorf, J. (2022a). Do students with specific learning disorders with impairments in reading benefit from linguistic simplification of test items in science? *Exceptional Children, 89*(1), 23–41. 10.1177/00144029221094

[CR26] Cruz Neri, N., Klückmann, F., & Retelsdorf, J. (2022b). LATIC–A linguistic analyzer for text and item characteristics. *PLOS One, 17*(11), e0277250. 10.1371/journal.pone.0277250 10.1371/journal.pone.0277250 PMC9632876 36327343

[CR27] Dascalu, M., Dessus, P., Trausan-Matu, Ş. B., & Nardy, A. (2013). ReaderBench, an environment for analyzing text complexity and reading strategies. In H. C. Lane, K. Yacef, J. Mostow, & P. Pavlik, *Proceedings of the 16th International Conference on Artificial Intelligence in Education (AIED 2013)* (pp. 379–388). Springer. 10.1007/978-3-642-39112-5_39

[CR28] Dodell-Feder, D., Koster-Hale, J., Bedny, M., & Saxe, R. (2011). fMRI item analysis in a theory of mind task. *NeuroImage, 55*(2), 705–712. 10.1016/j.neuroimage.2010.12.040 10.1016/j.neuroimage.2010.12.040 21182967

[CR29] Dryer, M. S., & Haspelmath, M. (2013). WALS Online (v2020.3) [Data set]. Zenodo. 10.5281/zenodo.7385533

[CR30] Dudău, D. P., & Sava, F. A. (2021). Performing multilingual analysis with linguistic inquiry and word count 2015 (LIWC2015). An equivalence study of four languages. *Frontiers in Psychology, 12*, 2860. 10.3389/fpsyg.2021.570568 10.3389/fpsyg.2021.570568 PMC8311520 34322047

[CR31] Eberhard, D. M., Simons, G. F., & Fennig, C. D. (2022). *Ethnologue: Languages of the world* ((25 ed.). ed.). SIL International.

[CR32] Evans, N., & Levinson, S. C. (2009). The myth of language universals: Language diversity and its importance for cognitive science. *Behavioral and Brain Sciences, 32*(5), 429–448. 10.1017/S0140525X0999094X 19857320 10.1017/S0140525X0999094X

[CR33] Faruqui, M., Tsvetkov, Y. R., & Dyer, C. (2016). Problems with evaluation of word embeddings using word similarity tasks. In O. Levy, F. Hill, A. Korhonen, K. Cho, R. Reichart, Y. Goldberg, & A. Bordes, *Proceedings of the 1st Workshop on Evaluating Vector-Space Representations for NLP* (pp. 30–35). Association for Computational Linguistics. 10.18653/v1/W16-2506

[CR34] Fortuna, P., & Nunes, S. (2018). A survey on automatic detection of hate speech in text. *ACM Computing Surveys, 51*(4), 85. 10.1145/3232676

[CR35] Francis, M. E., & Pennebaker, J. W. (1992). Putting stress into words: The impact of writing on physiological, absentee, and self-reported emotional well-being measures. *American Journal of Health Promotion, 6*(4), 280–287. 10.4278/0890-1171-6.4.280 10.4278/0890-1171-6.4.280 10146806

[CR36] Georgi, R., Xia, F., & Lewis, W. (2010). Comparing language similarity across genetic and typologically-based groupings. In C.-R. Huang, & D. Jurafsky, *Proceedings of the 23rd International Conference on Computational Linguistics* (pp. 385–393). Coling 2010 Organizing Committee.

[CR37] Gibson, E., Futrell, R., Piantadosi, S. P., Dautriche, I., Mahowald, K., Bergen, L., & Levy, R. (2019). How efficiency shapes human language. *Trends in Cognitive Sciences, 23*(5), 389–407. 10.1016/j.tics.2019.02.003 10.1016/j.tics.2019.02.003 31006626

[CR38] Gimenes, M., & New, B. (2016). Worldlex: Twitter and blog word frequencies for 66 languages. *Behavior Research Methods, 48*, 963–972. 10.3758/s13428-015-0621-0 10.3758/s13428-015-0621-0 26170053

[CR39] Graesser, A. C., McNamara, D. S., & Cai, Z. (2004). Coh-Metrix: Analysis of text on cohesion and language. *Behavior Research Methods, Instruments, & Computers, 36*(2), 193–202. 10.3758/BF03195564 10.3758/bf03195564 15354684

[CR40] Grave, E., Bojanowski, P., Gupta, P., Joulin, A., & Mikolov, T. (2018). Learning word vectors for 157 languages. In N. Calzolari, K. Choukri, C. Cieri, T. Declerck, S. Goggi, K. Hasida, H. Isahara, B. Maegaard, J. Mariani, H. Mazo, A. Moreno, J. Odijk, S. Piperidis, & T. Tokunaga, *Proceedings of the 11th International Conference on Language Resources and Evaluation (LREC'18)* (pp. 3483–3487). European Language Resources Association.

[CR41] Gualberto, E. S., De Sousa, R. T., Vieira, T. P., Da Costa, J. L. P. C., & Duque, C. G. (2020). The answer is in the text: multi-stage methods for phishing detection based on feature engineering. *IEEE Access, 8*, 223529–223547. 10.1109/ACCESS.2020.3043396

[CR42] Gutu-Robu, G., Sirbu, M.-D. P., Dascălu, M., Dessus, P., & Trausan-Matu, S. (2018). Liftoff–ReaderBench introduces new online functionalities. *Romanian Journal of Human–Computer Interaction, 11*(1), 76–91.

[CR43] Hart, R. P. (2017). Diction (software). *The International Encyclopedia of Communication Research Methods*, 1–2. 10.1002/9781118901731.iecrm0066

[CR44] Henrich, J., Heine, S. J., & Norenzayan, A. (2010). The weirdest people in the world? *Behavioral and Brain Sciences, 33*(2–3), 61–83. 10.1017/S0140525X0999152X 10.1017/S0140525X0999152X 20550733

[CR45] Juola, P. (2008). Authorship attribution. *Foundations and Trends in Information Retrieval, 1*(3), 233–334. 10.1561/1500000005

[CR46] Kim, E.-K., & Jo, H.-H. (2016). Measuring burstiness for finite event sequences. *Physical Review E, 94*(3), 032311. 10.1103/PhysRevE.94.032311 10.1103/PhysRevE.94.032311 27739800

[CR47] Kučera, D., & Mehl, M. R. (2022). Beyond English: Considering language and culture in psychological text analysis. *Frontiers in Psychology, 13*, 819543. 10.3389/fpsyg.2022.819543 35310262 10.3389/fpsyg.2022.819543 PMC8931497

[CR48] Kyle, K. (2016). *Measuring syntactic development in L2 writing: Fine grained indices of syntactic complexity and usage-based indices of syntactic sophistication.* Georgia State University. 10.57709/8501051

[CR49] Landauer, T. K., McNamara, D. S., Dennis, S., & Kintsch, W. (2007). *Handbook of latent semantic analysis.* Lawrence Erlbaum Associates.

[CR50] Laur, S., Orasmaa, S., Särg, D., & Tammo, P. (2020). EstNLTK 1.6: Remastered Estonian NLP pipeline. In N. Calzolari, F. Béchet, P. Blache, K. Choukri, C. Cieri, T. Declerck, S. Goggi, H. Isahara, B. Maegaard, J. Mariani, H. Mazo, A. Moreno, J. Odijk, & S. Piperidis, *Proceedings of the 12th Language Resources and Evaluation Conference (LREC'20)* (pp. 7152–7160). European Language Resources Association.

[CR51] Lauriola, I., Lavelli, A., & Aiolli, F. (2022). An introduction to deep learning in natural language processing: Models, techniques, and tools. *Neurocomputing, 470*, 443–456. 10.1016/j.neucom.2021.05.103 10.1016/j.neucom.2021.05.103

[CR52] Levenshtein, V. I. (1966). Binary codes capable of correcting deletions, insertions, and reversals. *Soviet Physics Doklady, 10*(8), 707–710.

[CR53] Levisen, C. (2019). Biases we live by: Anglocentrism in linguistics and cognitive sciences. *Language Sciences, 76*, 101173. 10.1016/j.langsci.2018.05.010 10.1016/j.langsci.2018.05.010

[CR54] Levshina, N. (2019). Token-based typology and word order entropy: A study based on Universal Dependencies. *Linguistic Typology, 23*(3), 533–572. 10.1515/lingty-2019-0025 10.1515/lingty-2019-0025

[CR55] Li, X., Huang, L., Yao, P., & Hyönä, J. (2022). Universal and specific reading mechanisms across different writing systems. *Nature Reviews Psychology, 1*(3), 133–144. 10.1038/s44159-022-00022-6 10.1038/s44159-022-00022-6

[CR56] Linders, G. M., & Louwerse, M. M. (2023). Zipf’s law revisited: Spoken dialog, linguistic units, parameters, and the principle of least effort. *Psychonomic Bulletin & Review, 30*, 77–101. 10.3758/s13423-022-02142-9 35840837 10.3758/s13423-022-02142-9 PMC9971120

[CR57] Louwerse, M. M. (2004). Semantic variation in idiolect and sociolect: Corpus linguistic evidence from literary texts. *Computers and the Humanities, 38*, 207–221. 10.1023/B:CHUM.0000031185.88395.b1 10.1023/B:CHUM.0000031185.88395.b1

[CR58] Louwerse, M. M. (2011). Symbol interdependency in symbolic and embodied cognition. *Topics in Cognitive Science, 3*(2), 273–302. 10.1111/j.1756-8765.2010.01106.x 25164297 10.1111/j.1756-8765.2010.01106.x

[CR59] Louwerse, M. M. (2018). Knowing the meaning of a word by the linguistic and perceptual company it keeps. *Topics in Cognitive Science, 10*(3), 573–589. 10.1111/tops.12349 29851286 10.1111/tops.12349

[CR60] Louwerse, M. M. (2021). *Keeping those words in mind: How language creates meaning*. Rowman & Littlefield.

[CR61] Louwerse, M. M., McCarthy, P. M., McNamara, D. S., & Graesser, A. C. (2004). Variation in language and cohesion across written and spoken registers. In K. D. Forbus, D. Gentner, & T. Regier, *Proceedings of the 26th Annual Meeting of the Cognitive Science Society* (pp. 843–848).

[CR62] Lu, X. (2010). Automatic analysis of syntactic complexity in second language writing. *International Journal of Corpus Linguistics, 15*(4), 474–496. 10.1075/ijcl.15.4.02lu 10.1075/ijcl.15.4.02lu

[CR63] Lupyan, G., Rahman, R. A., Boroditsky, L., & Clark, A. (2020). Effects of language on visual perception. *Trends in Cognitive Sciences, 24*(11), 930–944. 10.1016/j.tics.2020.08.005 33012687 10.1016/j.tics.2020.08.005

[CR64] MacWhinney, B. (2007). The Talkbank project. In I. J. Beal, K. Corrigan, & H. Moisl (Eds.), *Creating and Digitizing Language Corpora: Volume 1: Synchronic Databases* (pp. 163–180). Palgrave Macmillan. 10.1057/9780230223936_7

[CR65] Magueresse, A., Carles, V., & Heetderks, E. (2020). Low-resource languages: A review of past work and future challenges. *arXiv preprint arXiv:2006.07264*. 10.48550/arXiv.2006.07264

[CR66] Malmasi, S., Evanini, K., Cahill, A., Tetreault, J., Pugh, R., Hamill, C., Napolitano, D., & Qian, Y. (2017). A report on the 2017 native language identification shared task. In J. Tetreault, J. Burstein, C. Leacock, & H. Yannakoudakis, *Proceedings of the 12th Workshop on Innovative Use of NLP for Building Educational Applications* (pp. 62–75). Association for Computational Linguistics. 10.18653/v1/W17-5007

[CR67] Maslennikova, A., Labruna, P., Cimino, A., & Dell'Orletta, F. (2019). Quanti anni hai? Age Identification for Italian. In R. Bernardi, R. Navigli, & G. Semeraro, *Proceedings of the Sixth Italian Conference on Computational Linguistics.* Italian Association for Computational Linguistics.

[CR68] Maynard, S. K. (1986). On back-channel behavior in Japanese and English casual conversation. *Linguistics, 24*(6), 1079–1108. 10.1515/ling.1986.24.6.1079 10.1515/ling.1986.24.6.1079

[CR69] McCarthy, P. M., Lewis, G. A., Dufty, D. F., & McNamara, D. S. (2006). Analyzing writing styles with Coh-Metrix. In G. Sutcliffe, & R. Goebel, *Proceedings of the Nineteenth International Florida Artificial Intelligence Research Society Conference* (pp. 764–769). AAAI Press.

[CR70] McNamara, D. S., Graesser, A. C., & Louwerse, M. M. (2012). Sources of text difficulty: Across genres and grades. In J. Sabatini, E. Albro, & T. O'Reilly, *Measuring up: Advances in how we assess reading ability* (pp. 89–116). Rowman & Littlefield.

[CR71] McNamara, D. S., Graesser, A. C., McCarthy, P. M., & Cai, Z. (2014). Automated evaluation of text and discourse with Coh-Metrix. Cambridge University Press. 10.1017/CBO9780511894664

[CR72] McNamara, D. S., Louwerse, M. M., McCarthy, P. M., & Graesser, A. C. (2010). Coh-Metrix: Capturing linguistic features of cohesion. *Discourse Processes, 47*(4), 292–330. 10.1080/01638530902959943 10.1080/01638530902959943

[CR73] McTavish, D. G., & Pirro, E. B. (1990). Contextual content analysis. *Quality & Quantity, 24*(3), 245–265. 10.1007/BF00139259 10.1007/BF00139259

[CR74] Miaschi, A., Brunato, D., Dell’Orletta, F., & Venturi, G. (2020). Linguistic profiling of a neural language model. In D. Scott, N. Bel, & C. Zong, *Proceedings of the 28th International Conference on Computational Linguistics* (pp. 745–756). International Committee on Computational Linguistics. 10.18653/v1/2020.coling-main.65

[CR75] Mihalcea, R., & Strapparava, C. (2009). The lie detector: Explorations in the automatic recognition of deceptive language. In K.-Y. Su, J. Su, J. Wiebe, & H. Li, *Proceedings of the Joint Conference of the 47th Annual Meeting of the Association for Computational Linguistics and 4th International Joint Conference on Natural Language Processing of the AFNLP: Short Papers* (pp. 309–312). Association for Computational Linguistics.

[CR76] Nini, A. (2019). The multi-dimensional analysis tagger. In T. B. Sardinha, & M. V. Pinto, *Multi-Dimensional Analysis: Research Methods and Current Issues* (pp. 67–94). Bloomsbury Academic. 10.5040/9781350023857.0012

[CR77] Nivre, J., de Marneffe, M.-C., Ginter, F., Hajič, J., Manning, C. D., Pyysalo, S., Schuster, S., Tyers, F., & Zeman, D. (2020). Universal Dependencies v2: An evergrowing multilingual treebank collection. In N. Calzolari, F. Béchet, P. Blache, K. Choukri, C. Cieri, T. Declerck, S. Goggi, H. Isahara, B. Maegaard, J. Mariani, H. Mazo, A. Moreno, J. Odijk, & S. Piperidis, *Proceedings of the 12th Language Resources and Evaluation Conference (LREC'20)* (pp. 4034–4043). European Language Resources Association.

[CR78] North, R., Lagerstrom, R., & Mitchell, W. (1972). *Diction computer program*. Inter-university Consortium for Political and Social Research.

[CR79] Obeid, O., Zalmout, N., Khalifa, S., Taji, D., Oudah, M., Alhafni, B., Inoue, G., Eryani, F., Erdmann, A., & Habash, N. (2020). CAMeL tools: An open source python toolkit for Arabic natural language processing. In N. Calzolari, F. Béchet, P. Blache, K. Choukri, C. Cieri, T. Declerck, S. Goggi, H. Isahara, B. Maegaard, J. Mariani, H. Mazo, A. Moreno, J. Odijk, & S. Piperidis, *Proceedings of the 12th Language Resources and Evaluation Conference (LREC'20)* (pp. 7022–7032). European Language Resources Association.

[CR80] OpenAI. (2023). ChatGPT (Mar 23 version) [Large language model]. Retrieved from https://chat.openai.com/

[CR81] Păiș, V., Ion, R., Avram, A.-M., & Mitrofan, M. T. (2021). In-depth evaluation of Romanian natural language processing pipelines. *Romanian Journal of Information Science and Technology, 24*(4), 384–401.

[CR82] Pander Maat, H., Kraf, R., van den Bosch, A., Dekker, N., van Gompel, M., Kleijn, S., Sanders, T., & van der Sloot, K. (2014). T-Scan: A new tool for analyzing Dutch text. *Computational Linguistics in the Netherlands Journal, 4*, 53–74.

[CR83] Pennebaker, J. W., & King, L. A. (1999). Linguistic styles: Language use as an individual difference. *Journal of Personality and Social Psychology, 77*(6), 1296–1312. 10.1037/0022-3514.77.6.1296 10626371 10.1037/0022-3514.77.6.1296

[CR84] Piantadosi, S. T., Tily, H., & Gibson, E. (2011). Word lengths are optimized for efficient communication. *Proceedings of the National Academy of Sciences, 108*(9), 3526–3529. 10.1073/pnas.1012551108 10.1073/pnas.1012551108 PMC3048148 21278332

[CR85] Qi, P., Zhang, Y., Zhang, Y., Bolton, J., & Manning, C. D. (2020). Stanza: A Python natural language processing toolkit for many human languages. In A. Celikyilmaz, & T.-H. Wen, *Proceedings of the 58th Annual Meeting of the Association for Computational Linguistics: System Demonstrations* (pp. 101–108). Association for Computational Linguistics. 10.18653/v1/2020.acl-demos.14

[CR86] Qiu, X., Zhang, Q., & Huang, X. (2013). FudanNLP: A toolkit for Chinese natural language processing. In M. Butt, & S. Hussain, *Proceedings of the 51st Annual Meeting of the Association for Computational Linguistics: System Demonstrations* (pp. 49–54). Association for Computational Linguistics.

[CR87] Rae, J. W., Borgeaud, S., Cai, T., Millican, K., Hoffmann, J., Song, F., Aslanides, J., Henderson, S., Ring, R., Young, S., Rutherford, E., Hennigan, T., Menick, J., Cassirer, A., & Irving, G. (2021). Scaling language models: Methods, analysis & insights from training Gopher. *arXiv preprint arXiv:2112.11446*. 10.48550/arXiv.2112.11446

[CR88] Rama, T., & Kolachina, P. (2012). How good are typological distances for determining genealogical relationships among languages? In M. Kay, & C. Boitet, *Proceedings of COLING 2012: Posters* (pp. 975–984). The COLING 2012 Organizing Committee.

[CR89] Rashkin, H., Choi, E., Jang, J. Y., Volkova, S., & Choi, Y. (2017). Truth of varying shades: Analyzing language in fake news and political fact-checking. In M. Palmer, R. Hwa, & S. Riedel, *Proceedings of the 2017 Conference on Empirical Methods in Natural Language Processing* (pp. 2931–2937). Association for Computational Linguistics. 10.18653/v1/D17-1317

[CR90] Reyes, A., Rosso, P., & Buscaldi, D. (2012). From humor recognition to irony detection: The figurative language of social media. *Data & Knowledge Engineering, 74*, 1–12. 10.1016/j.datak.2012.02.005 10.1016/j.datak.2012.02.005

[CR91] Roberts, C. W. (2000). A conceptual framework for quantitative text analysis. *Quality and Quantity, 34*(3), 259–274. 10.1023/A:1004780007748 10.1023/A:1004780007748

[CR92] Sadvilkar, N., & Neumann, M. (2020). PySBD: Pragmatic sentence boundary disambiguation. In E. L. Park, M. Hagiwara, D. Milajevs, N. F. Liu, G. Chauhan, & L. Tan, *Proceedings of Second Workshop for NLP Open Source Software* (pp. 110–114). Association for Computational Linguistics. 10.18653/v1/2020.nlposs-1.15

[CR93] Sarker, S. (2021). BNLP: Natural language processing toolkit for Bengali. *arXiv preprint arXiv:2102.00405*. 10.48550/arXiv.2102.00405

[CR94] Scarton, C., & Aluísio, S. M. (2010). Coh-Metrix-Port: A readability assessment tool for texts in Brazilian Portuguese. In *Proceedings of the 9th International Conference on Computational Processing of the Portuguese Language, Extended Activities Proceedings, PROPOR* (Vol. 10, pp. 1–2).

[CR95] Schler, J., Koppel, M., Argamon, S., & Pennebaker, J. W. (2005). Effects of age and gender on blogging. In I. N. Nicolov, F. Salvetti, M. Liberman, & J. H. Martin (Eds.), *Computational Approaches to Analyzing Weblogs: Papers from the AAAI Spring Symposium* (Vol. 6, pp. 199–205). AAAI Press.

[CR96] Schnabel, T., Labutov, I., Mimno, D., & Joachims, T. (2015). Evaluation methods for unsupervised word embeddings. In L. Màrquez, C. Callison-Burch, & J. Su, *Proceedings of the 2015 Conference on Empirical Methods in Natural Language Processing* (pp. 298–307). Association for Computational Linguistics. 10.18653/v1/D15-1036

[CR97] Share, D. L. (2008). On the Anglocentricities of current reading research and practice: The perils of overreliance on an "outlier" orthography. *Psychological Bulletin, 134*(4), 584–615. 10.1037/0033-2909.134.4.584 18605821 10.1037/0033-2909.134.4.584

[CR98] Sirts, K., & Peekman, K. (2020). Evaluating sentence segmentation and word tokenization systems on Estonian web texts. In A. Utka, J. Vaičenonienė, J. Kovalevskaitė, & D. Kalinauskaitė, *Proceedings of the Ninth International Conference Baltic Human Language Technologies* (pp. 174–181). IOS Press.

[CR99] Sokal, R. R., & Rohlf, F. J. (1962). The comparison of dendrograms by objective methods. *Taxon, 11*(2), 33–40. 10.2307/1217208 10.2307/1217208

[CR100] Stolcke, A., Ries, K., Coccaro, N., Shriberg, E., Bates, R., Jurafsky, D., Taylor, P., Martin, R., Van Ess-Dykema, C., & Meteer, M. (2000). Dialogue act modeling for automatic tagging and recognition of conversational speech. *Computational Linguistics, 26*(3), 339–373. 10.1162/089120100561737 10.1162/089120100561737

[CR101] Straka, M., & Straková, J. (2017). Tokenizing, POS tagging, lemmatizing and parsing UD 2.0 with UDPipe. In J. Hajič, & D. Zeman, *Proceedings of the CoNLL 2017 Shared Task: Multilingual Parsing from Raw Text to Universal Dependencies* (pp. 88–99). Association for Computational Linguistics. 10.18653/v1/K17-3009

[CR102] Straka, M., Hajic, J., & Straková, J. (2016). UDPipe: Trainable pipeline for processing CoNLL-U files performing tokenization, morphological analysis, POS tagging and parsing. In N. Calzolari, K. Choukri, T. Declerck, S. Goggi, M. Grobelnik, B. Maegaard, J. Mariani, H. Mazo, A. Moreno, J. Odijk, & S. Piperidis, *Proceedings of the 10th International Conference on Language Resources and Evaluation (LREC'16)* (pp. 4290–4297). European Language Resources Association.

[CR103] Tausczik, Y. R., & Pennebaker, J. W. (2010). The psychological meaning of words: LIWC and computerized text analysis methods. *Journal of Language and Social Psychology, 29*(1), 24–54. 10.1177/0261927X09351676 10.1177/0261927X09351676

[CR104] Thelwall, M., Buckley, K., Paltoglou, G., Cai, D., & Kappas, A. (2010). Sentiment strength detection in short informal text. *Journal of the American Society for Information Science and Technology, 61*(12), 2544–2558. 10.1002/asi.21416 10.1002/asi.21416

[CR105] Trevisan, P., & García, A. M. (2019). Systemic functional grammar as a tool for experimental stimulus design: New appliable horizons in psycholinguistics and neurolinguistics. *Language Sciences, 75*, 35–46. 10.1016/j.langsci.2019.101237 10.1016/j.langsci.2019.101237

[CR106] Tuckute, G., Sathe, A., Wang, M., Yoder, H., & Shain, C. F. (2022). SentSpace: Large-scale benchmarking and evaluation of text using cognitively motivated lexical, syntactic, and semantic features. In H. Hajishirzi, Q. Ning, & A. Sil, *Proceedings of the 2022 Conference of the North American Chapter of the Association for Computational Linguistics: Human Language Technologies: System Demonstrations* (pp. 99–113). Association for Computational Linguistics. 10.18653/v1/2022.naacl-demo.11

[CR107] Türkoğlu, F., Diri, B., & Amasyalı, M. F. (2007). Author attribution of Turkish texts by feature mining. In I. D.-S. Huang, L. Heutte, & M. Loog (Eds.), *Advanced Intelligent Computing Theories and Applications: With Aspects of Theoretical and Methodological Issues* (pp. 1086–1093). Springer. 10.1007/978-3-540-74171-8

[CR108] Van Heuven, W. J., Mandera, P., Keuleers, E., & Brysbaert, M. (2014). SUBTLEX-UK: A new and improved word frequency database for British English. *Quarterly Journal of Experimental Psychology, 67*(6), 1176–1190. 10.1080/17470218.2013.850521 10.1080/17470218.2013.850521 24417251

[CR109] Van Wissen, L., & Boot, P. (2017). An electronic translation of the LIWC dictionary into Dutch. In I. Kosem, C. Tiberius, M. Jakubíček, J. Kallas, S. Krek, & V. Baisa, *Electronic lexicography in the 21st century. Proceedings of the eLex 2017 Conference.* (pp. 703–715). Lexical Computing CZ.

[CR110] Wang, B., Wang, A., Chen, F. W., & Kuo, C.-C. J. (2019). Evaluating word embedding models: Methods and experimental results. *APSIPA Transactions on Signal and Information Processing, 8*, e19. 10.1017/ATSIP.2019.12 10.1017/ATSIP.2019.12

[CR111] Wichmann, S., Holman, E. W., Bakker, D., & Brown, C. H. (2010). Evaluating linguistic distance measures. *Physica A: Statistical Mechanics and its Applications, 389*(17), 3632–3639. 10.1016/j.physa.2010.05.011 10.1016/j.physa.2010.05.011

[CR112] Zellers, M. (2021). An overview of forms, functions, and configurations of backchannels in Ruruuli/Lunyala. *Journal of Pragmatics, 175*, 38–52. 10.1016/j.pragma.2021.01.012 10.1016/j.pragma.2021.01.012

[CR113] Zipf, G. K. (1949). *Human behavior and the principle of least effort*. Addison-Wesley.

