# Publisher Correction: Lingualyzer: A computational linguistic tool for multilingual and multidimensional text analysis

**DOI:** 10.3758/s13428-023-02314-y

**Published:** 2023-12-13

**Authors:** Guido M. Linders, Max M. Louwerse

**Affiliations:** 1 Department of Cognitive Science & Artificial Intelligence, Tilburg University, Tilburg, Netherlands; 2 Department of Comparative Language Science, University of Zurich, Zurich, Switzerland

**Publisher Correction: Behavior Research Methods**

10.3758/s13428-023-02284-1

The original online version of this article was revised: a typographical error was made in the references section.

In the reference, Cruz Neri, N., Klückmann, F., & Retelsdorf, J. (2022). LATIC–A linguistic analyzer for text and item characteristics. PLOS One, 17(11), e0277250. Florian Klückmann is missing, and his initials are instead added to the initials of the first author: Cruz Neri, N. K. F., & Retelsdorf, J. (2022b). This has been corrected.

